# Engineering p–d Coupling at Fe–Bi and Zn–Bi Sites for Efficient Li–S Conversion

**DOI:** 10.1021/acs.nanolett.5c04356

**Published:** 2025-11-05

**Authors:** Jing Yu, Zhifu Liang, Xianggui Zhou, Chaoqi Zhang, Ivan Pinto-Huguet, Oleg Usoltsev, Chao Yue Zhang, Alex W. Robertson, Andreu Cabot, Jordi Arbiol

**Affiliations:** † Catalonia Institute for Energy Research (IREC), Sant Adrià de Besòs, Barcelona 08930, Catalonia, Spain; ‡ Catalan Institute of Nanoscience and Nanotechnology (ICN2), CSIC and BIST, Campus UAB, Bellaterra, 08193 Barcelona, Catalonia, Spain; § College of Ocean Science and Engineering, 12477 Shanghai Maritime University, 201306 Shanghai, China; ∥ South China Advanced Institute for Soft Matter Science and Technology, School of Emergent Soft Matter, 26467 South China University of Technology, Guangzhou 510640, China; ⊥ College of Materials Science and Engineering, 12423 Fuzhou University, No.2, Xueyuan Road, Minhou County, Fuzhou City, Fujian Province 350108, China; # CELLS-ALBA Synchrotron Carrer de la Llum, 2, 26, 08290 Cerdanyola del Vallès, Barcelona, Catalonia, Spain; ∇ Department of Chemistry and Biochemistry, 8783 University of California, Los Angeles, California 90095, United States; ○ Department of Physics, University of Warwick, Coventry CV4 7AL, U.K.; ● ICREA, Pg. Lluis Companys, Barcelona 08010, Catalonia, Spain

**Keywords:** Dual-atom catalyst, polysulfide, lithium−sulfur battery, sulfur cathode, single-atom catalyst

## Abstract

Electrocatalytic sulfur hosts are crucial for high-performance, lightweight lithium–sulfur (Li–S) batteries, demanding atomically dispersed metals with precisely tuned electronic structures. Here, we present 3d–6p dual-atom catalysts (DACs) composed of earth-abundant Fe–Bi or Zn–Bi pairs anchored on nitrogen-doped carbon. Aberration-corrected scanning transmission electron microscopy and X-ray absorption spectroscopy confirm atomically dispersed bimetallic centers stabilized through TM-N coordination. In Li–S cells, DACs, particularly Fe–Bi, markedly accelerate polysulfide conversion, achieving near-ideal discharge plateau ratios, superior rate capability with >50% capacity retention at 3C, and about 80% retention after 1000 cycles at 1C. Density functional theory indicates that Bi induces strong ligand-field and p–d coupling effects, shifting Fe 3d states toward the Fermi level and polarizing Zn through charge redistribution. This Bi-driven cooperative activation transcends d-electron limitations, offering a general and scalable route to high-rate, durable Li–S electrocatalysts.

## Graphical abstract

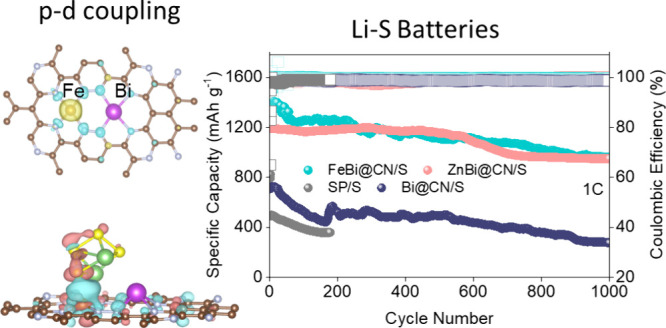

The accelerated transition toward sustainable energy requires storage technologies that surpass the limitations of conventional lithium-ion batteries, whose reliance on critical raw materials such as nickel and cobalt raises both cost and sustainability concerns. Lithium–sulfur (Li–S) batteries are among the most promising next-generation candidates, benefiting from the abundance of sulfur and its exceptionally high theoretical specific capacity (1675 mAh g^–1^).^1^ However, their commercialization is hindered by the dissolution and migration of lithium polysulfides (LiPS), which induce rapid self-discharge, low energy efficiency, and limited cycle life.^2–5^

To mitigate these challenges, a wide range of strategies, including physical confinement, chemical anchoring, electrolyte optimization, and redox catalysis, have been investigated.^6–8^ Among them, catalytic approaches are particularly effective in accelerating polysulfide conversion and improving cell stability. Yet, many reported catalysts still rely on scarce and expensive compounds, especially Co-based materials, which compromises scalability.^9–12^

Recent efforts have focused on atomically dispersed transition metal (TM) catalysts. By maximizing atom utilization, they reduce cost and weight while providing well-defined, uniform active sites.^13–15^ Nonetheless, the single-site nature of these systems restricts their tunability and multifunctionality. Dual-atom catalysts, which integrate two distinct metals in close proximity, address this limitation by offering cooperative electronic effects, an expanded design space, and enhanced catalytic performance.^16–18^

Beyond TMs, p-block elements, most notably bismuth (Bi), have emerged as unconventional yet promising catalytic centers. Bi is an earth-abundant, air-stable semimetal with high electrical conductivity and a 6s^2^6p^3^ valence configuration.^19^ Although it lacks d orbitals, the highly polarizable 6p orbitals of Bi interact strongly with sulfur 3p states, enabling p–p coupling and electron delocalization that mimic d-block catalysis.^20,21^ Furthermore, large Bi­(III) centers exhibit borderline-soft Lewis acidity and strong polarizability, features that enhance LiPS adsorption, facilitate charge transfer, weaken S–S bonds, and promote product desorption, altogether lowering reaction barriers.^20^ A persistent challenge, however, lies in preventing Bi aggregation, which reduces the density of accessible active sites and compromises durability.

In this work, we address these limitations by anchoring Bi as atomically dispersed centers within a nitrogen-doped carbon (CN) scaffold. The CN matrix provides robust N-coordination to stabilize isolated Bi sites, while its conductivity and porosity ensure efficient charge/ion transport and favorable reaction kinetics. To further augment catalytic performance, we incorporate 3d TMs, particularly iron (Fe) and zinc (Zn), to form dual-atom TM–Bi catalysts. This selection leverages earth-abundant, electrochemically stable metals with contrasting electronic structures: Fe, with a partially filled 3d^6^ manifold and high density of states near the Fermi level, can engage in redox-coupled electron transfer, whereas Zn, with a closed-shell 3d^10^ configuration, is comparatively inert. This contrast provides a platform to probe ligand-field-induced activation of Bi and to explore cooperative site-to-site synergy in polysulfide catalysis, as shown in Figure 1a.

**Figure 1:**
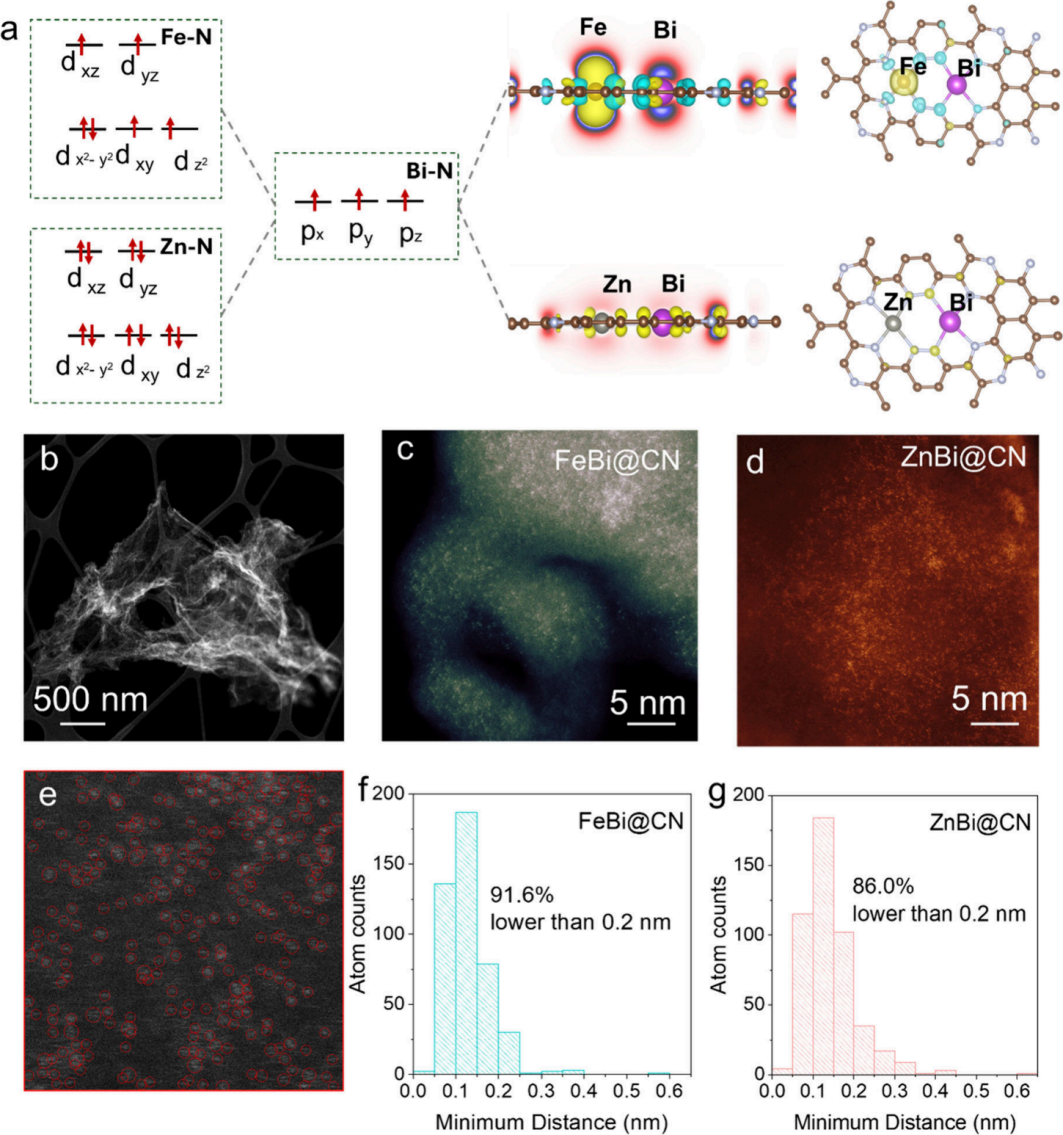
(a) Material design concept and electronic structure schematic of FeBi@CN and ZnBi@CN DACs. (b) HAADF-STEM image of FeBi@CN. (c, d) False-color atomic-resolution AC HAADF-STEM images of FeBi@CN (c) and ZnBi@CN (d). (e) AC HAADF STEM images of Zn–Bi/CN marking atomic pairs. Atom counting = 7 atoms nm^–2^. (f, g) Histograms of minimum interatomic distances between metal atoms in FeBi@CN and ZnBi@CN, showing that 91.6% for FeBi@CN and 86.0% for ZnBi@CN of atom pairs exhibit separations below 0.2 nm.

Fe–Bi and Zn–Bi atomic pairs supported on CN (FeBi@CN and ZnBi@CN) were prepared via a precoordination–pyrolysis route (see details in the Supporting Information, SI). Briefly, under argon and ice-bath cooling, chloroanilic acid was dissolved in *N*-methyl-2-pyrrolidone (NMP) in a three-neck flask. Melamine, BiCl_3_, and an equimolar amount of FeCl_2_ or ZnCl_2_ were added at 0 °C. With vigorous stirring, several drops of concentrated H_2_SO_4_ were introduced slowly. After 20 min, the mixture was warmed to room temperature and then heated to 170 °C for 24 h under argon. The resulting solid was cooled, vacuum-filtered, washed sequentially with ethanol and water, and freeze-dried for 24 h. Finally, the black powder was annealed at 700 °C for 3 h under argon to yield FeBi@CN or ZnBi@CN.

High angle annular dark field scanning transmission electron microscopy– (HAADF STEM) imaging (Figure 1b) of the DACs reveals an irregular, porous, flocculent architecture, while energy-dispersive X-ray spectroscopy (EDS) mapping confirms the homogeneous distribution of C, N, Fe/Zn, and Bi (Figures S1 and S2). Inductively coupled plasma optical emission spectrometry (ICP-OES) places the total metal loading at ∼1 wt %, with near-equal amounts of the TM and Bi (Table S1). Extensive HAADF STEM analyses detected no metal nanoparticles or clusters. Instead, aberration-corrected (AC) HAADF STEM (Figures 1c,d) show Fe/Zn and Bi uniformly anchored as atomically dispersed species on the CN matrix, with a high density of isolated single atoms, quantified at 10 ± 3 atoms nm^–2^ (Figures 1e-g and S3,S4).

Most (>85%) metal centers are observed to form atomic pairs (Figure S4), exhibiting an average nearest-neighbor distance of ∼1.2 Å (Figure S5). Density functional theory (DFT) calculations indicate comparable formation energies for Bi–Fe and Bi–Zn pairs relative to Bi–Bi, Fe–Fe, and Zn–Zn (Figure S6). Coupled with the equimolar metal composition, this supports a statistically equiprobable formation of heteroatomic (TM–Bi) and homoatomic (TM–TM, Bi–Bi) pairs, yielding an approximate ∼50% fraction of TM–Bi pairs.

Nitrogen adsorption–desorption isotherms show that the CN scaffold possesses a Brunauer–Emmett–Teller (BET) surface area of 115 m^2^ g^–1^ and a predominantly mesoporous structure with a pore size centered at ca. 1 nm (Figure S7). These nanopores can effectively trap sulfur within the carbon matrix, promoting intimate contact with catalytic sites and supporting rapid charge transport.

Survey X-ray photoelectron spectroscopy (XPS) confirms the presence of C, N, Fe/Zn, and Bi on the CN matrix in both FeBi@CN and ZnBi@CN (Figures 2a and S8). Owing to the low Fe, Zn, and Bi loadings, the metal signals are too weak for reliable oxidation-state assignment even in high-resolution spectra (Figures S9–S11). To overcome this limitation, synchrotron X-ray absorption spectroscopy (XAS) was employed to probe local coordination. Clear absorption features at the Fe, Zn, and Bi edges verify successful incorporation of these species into CN. Extended X-ray absorption fine structure (EXAFS) analysis at the Fe and Zn K-edges reveals dominant TM–N coordination with fitted bond lengths of ∼2.05 Å (Fe–N) and ∼2.02 Å (Zn–N) (Figure 2b–e), values characteristic of M–N_*x*_ sites. Subtle bond-length perturbations attributable to neighboring Bi are consistent with the presence of Bi–Fe and Bi–Zn dual-atom motifs, in agreement with microscopy evidence and DFT calculations.

**Figure 2:**
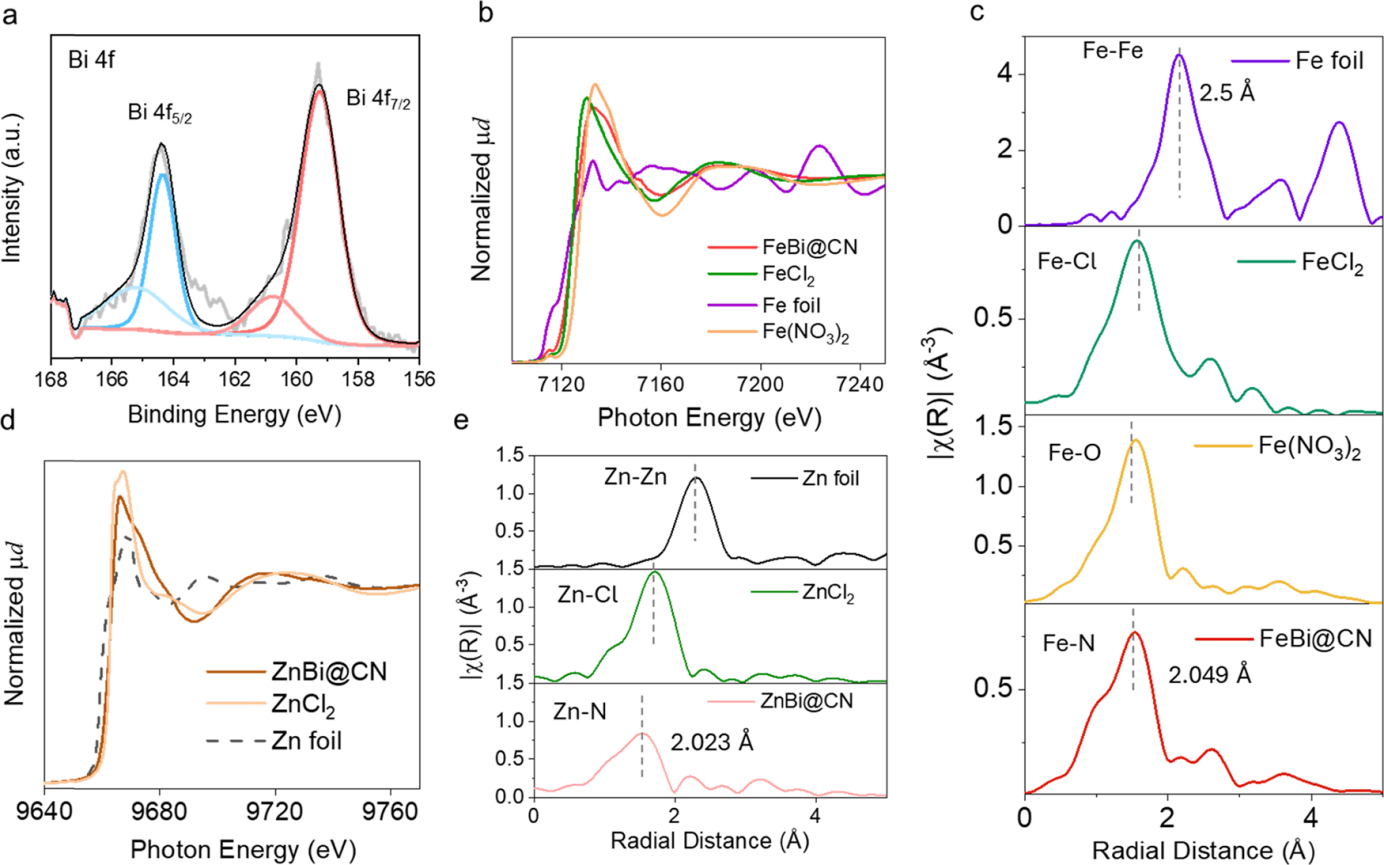
(a) High-resolution Bi 4f XPS spectrum of FeBi@CN. (b) Fe K-edge XANES spectra. (c) FT-EXAFS spectra of FeBi@CN, compared with reference samples including Fe foil, FeCl_2_, and Fe­(NO_3_)_2_. (d) Zn K-edge XANES spectra. (e) FT-EXAFS spectra of ZnBi@CN, compared with reference samples Zn foil and ZnCl_2_.

Symmetric cell cyclic voltammetry (CV) was employed to evaluate the catalytic activity of FeBi@CN and ZnBi@CN toward sulfur redox reactions (Figures S12 and S13). Symmetric cells were assembled using FeBi@CN or ZnBi@CN as both anode and cathode and using a DME/DOL mixture (1:1 v/v) electrolyte containing Li_2_S_6_ (see details in the SI). In these cells, the current response predominantly arises from the electrochemical conversion of polysulfides in the electrolyte. Specifically, Peak I corresponds to the conversion of Li_2_S_6_ to Li_2_S_4_ at one electrode, while Peak II corresponds to the subsequent conversion of Li_2_S_4_ to Li_2_S/Li_2_S_2_. FeBi@CN displays well-defined cathodic and anodic peaks within the potential window of −1.5 to 1.5 V, indicating efficient and highly reversible sulfur conversion. The peak positions shift with increasing scan rates (1–10 mV s^–1^). At low scan rates, sharp peaks indicate fast kinetics and high reversibility, whereas at higher scan rates, peak broadening reflects increased polarization and decreased redox efficiency. Compared with FeBi@CN, ZnBi@CN exhibits much broader and lower intensity peaks at all scan rates, with a markedly higher polarization and a near disappearance of Peak II at high scan rates, consistent with slower electron transfer and lower catalytic activity.

To evaluate the electrocatalytic activity of FeBi@CN and ZnBi@CN, CR2032 coin cells were assembled using these materials as catalytic sulfur hosts. Cathodes were fabricated by slurry-casting a mixture of TM–Bi/CN: Super P: PVDF = 8:1:1 (w/w/w) onto Al foil; elemental sulfur was then introduced by melt infusion to yield FeBi@CN/S and ZnBi@CN/S. S loading was set at ∼1.2 mg cm^–2^. All cells employed a lithium–metal counter/reference, a standard ether electrolyte (1.0 M LiTFSI and 0.2 M LiNO_3_ in DOL/DME, v/v). Electrochemical testing was conducted at C-rates referenced to 1675 mA g^–1^ (1 C) over a voltage window of 1.7–2.8 V vs Li^+^/Li.

Rate capability testing shows that FeBi@CN/S and ZnBi@CN/S retain ≈50.8% and ≈40.0% of their initial capacities, respectively, upon increasing the rate from 0.1C to 3C, substantially outperforming the Super-P and Bi@CN control (SP/S ∼16.8%, Bi@CN/S ∼31.4% retention; Figures 3a–c and Figures S14 and S15). Galvanostatic charge–discharge (GCD) curves exhibit the canonical two-plateau sulfur redox profile, with an initial plateau near ∼2.38 V and a second plateau below ∼2.1 V at 0.1C, indicative of efficient LiPS conversion.

**Figure 3:**
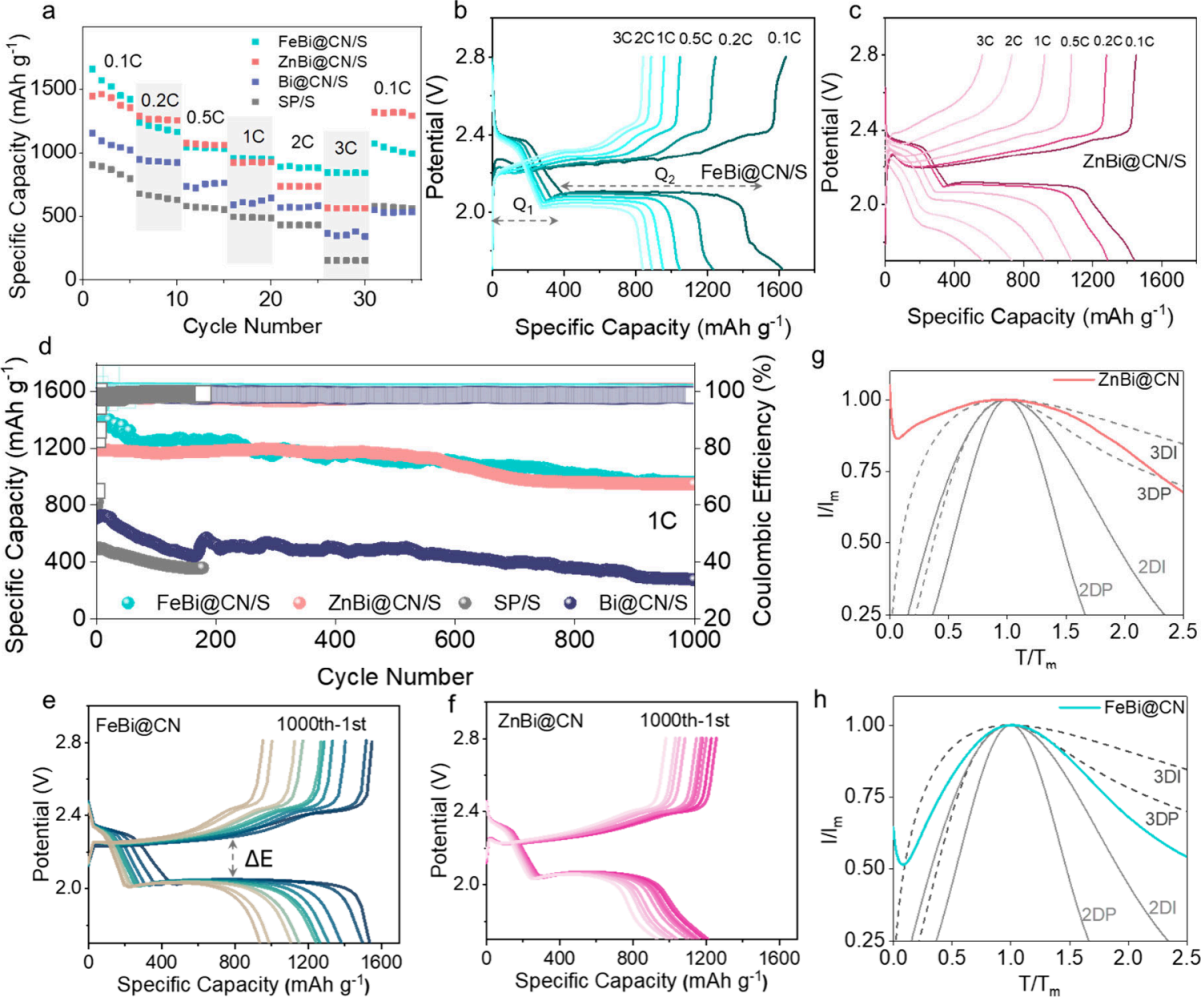
(a) Rate performance of Li–S cells at current rates from 0.1C to 3C. (b, c) GCD profiles at different rates (0.1C, 0.2C, 0.5C, 1C, 2C, and 3C) for FeBi@CN/S (b) and ZnBi@CN/S (c). (d) Long-term cycling performance at 1C of FeBi@CN/S, ZnBi@CN/S, Bi@CN/S, and SP/S cells. (e, f) GCD profiles at 1C for FeBi@CN/S (e) and ZnBi@CN/S (f) for over 1000 cycles. (g,h) Normalized t/t_m_ and (I/I_m_)^2^ plots (solid lines) compared with theoretical 3D instantaneous (3DI), 3D progressive (3DP), 2D instantaneous (2DI), and 2D progressive (2DP) nucleation models for ZnBi@CN and FeBi@CN electrodes.

To quantify catalytic efficacy, the capacity ratio of the second to first discharge plateau (Q_2_/Q_1_) was used: Q_1_ corresponds to S_8_ → long-chain LiPS, and Q_2_ to LiPS → Li_2_S_2_/Li_2_S. Although the theoretical Q_2_/Q_1_ is 3, experimental values are often reduced by competing chemical reactions, LiPS shuttling, and incomplete solid-state conversion. At 0.1C, FeBi@CN/S and ZnBi@CN/S deliver Q_2_/Q_1_ ≈ 2.98 and 2.97, respectively, versus ∼2.31 for Bi@CN/S and ∼1.90 for SP/S; at 3C, FeBi@CN/S and ZnBi@CN/S maintain ∼2.06 and ∼1.82, far above Bi@CN/S (∼0.73) and SP/S (∼0.81), underscoring accelerated LiPS conversion (Figure S16).

Among the two DACs, FeBi@CN/S exhibits a much stronger rate performance, reflected in higher capacity retention and a smaller increase in polarization/overpotential with current. Specifically, the voltage gap (ΔE) grows from 0.169 V at 0.1C to 0.305 V at 3C for FeBi@CN/S, compared with 0.137 V → 0.592 V for ZnBi@CN/S, indicating lower kinetic penalties for FeBi@CN/S at high rates (Table S3).

Collectively, these results demonstrate that asymmetric TM–Bi coordination sites in the CN scaffold effectively catalyze polysulfide conversion, enhancing reaction kinetics and reducing overpotentials, with FeBi@CN delivering the highest activity among the architectures evaluated.

Long-term cycling at 1C highlights the robustness of the DACs. After 50 activation cycles, FeBi@CN/S retains 76% of its initial capacity over the later 950 cycles (average decay ∼0.026% per cycle), while ZnBi@CN/S retains 81% over the same period (≈ 0.021% per cycle). By contrast, the Bi@CN/S control falls to 39% (∼0.061% per cycle) and SP/S to 46% after only 200 cycles (∼0.39% per cycle; Figure 3d), underscoring the superior stability imparted by Fe–Bi and Zn–Bi architectures.

The GCD curves remain well-defined after extensive cycling (Figures 3e,f and Figures S17 and S18). Even after 1000 cycles, FeBi@CN/S and ZnBi@CN/S maintain clear two-plateau behavior, with Q_2_ capacities of 719 and 686 mAh g^–1^, respectively, indicating sustained catalytic activity and structural integrity. In contrast, Bi@CN/S can only retain 200 mAh g^–1^ for Q_2_ capacities, while SP/S exhibits significant fading; the ∼2.1 V plateau associated with the Li_2_S_2_/Li_2_S formation is truncated by 200 cycles, contributing only ∼230 mAh g^–1^.

To further examine the catalytic effects, we conducted potentiostatic Li_2_S_2_/Li_2_S nucleation following galvanostatic discharge to 2.06 V (2.56 mA cm^–2^), with a potential hold at 2.05 V, using a 0.5 M Li_2_S_6_ + 1.0 M LiTFSI solution in DOL/DME (1:1 v/v) and FeBi@CN/S or ZnBi@CN/S cathodes with Li metal anodes. Upon nuclei formation, subsequent Li_2_S growth is governed by LiPS diffusion.^22^ Catalysts with strong polysulfide affinity promote surface diffusion of LiPSs, favoring two-dimensional (2D) Li_2_S growth, whereas catalysts with weaker polysulfide adsorption enable LiPS bulk diffusion, resulting in a three-dimensional (3D) Li_2_S growth. This divergence in nucleation and growth mechanisms leads to different contributions to the capacity in the second discharge plateau. Analysis of the nucleation mechanism (Figures 3g,h and Figure S19) reveals that FeBi@CN, ZnBi@CN, Bi@CN, and CN catalysts exhibit distinct nucleation behaviors. FeBi@CN exhibits the sharpest nucleation curves, indicative of a near-2D nucleation mechanism, whereas ZnBi@CN and Bi@CN trend toward 3D nucleation, and CN shows a broad peak consistent with pure 3D growth. These findings confirm that FeBi@CN effectively promotes sulfide precipitation from polysulfide intermediates, consistent with its accelerated LiPS conversion kinetics.

Cyclic voltammetry measurements reveal that the FeBi@CN/S sample exhibits the highest current response at 2.0 V, accompanied by a sharper peak, indicative of reduced polarization. In contrast, ZnBi@CN/S (1.9 V) and Bi@CN (1.87 V) display progressively lower peak currents and more pronounced polarization. The same trend is also observed at the oxidation peak (Figure S20). The Nyquist plots obtained from the electrochemical impedance spectroscopy (EIS) measurements of the various cathodes exhibit a semicircle in the high-frequency region and a straight line in the low-frequency region (Figure S21). The semicircle at high frequency corresponds to the charge-transfer resistance, whereas the inclined line at low frequency is associated with ion diffusion resistance. Among all the tested electrodes, FeBi@CN/S displays the smallest semicircle and the steepest low-frequency slope, indicating the lowest charge-transfer and diffusion resistances.

Overall, the electrochemical data show that the two TM-Bi DACs, FeBi@CN and ZnBi@CN, substantially enhance sulfur redox kinetics relative to the Super-P control. Among them, FeBi@CN delivers superior rate capability with lower polarization at high C-rates, consistent with faster charge-transfer–limited steps, whereas ZnBi@CN affords notable long-term capacity retention, also indicating excellent structural/chemical stability and persistent regulation of LiPS chemistry.

To rationalize these trends, DFT calculations were performed to examine the electronic structure, charge redistribution, and adsorption/reactivity of key LiPS intermediates at the Fe–Bi and Zn–Bi sites embedded within the CN matrix.

DFT results indicate that Bi reconstructs the local electronic structure at Fe: The Fe 3d states shift toward the Fermi level, increasing electron-donation capacity and redox reactivity; concomitant spin-state modulation further enhances the multielectron transfer capability of the Fe center (Figure S22). These features make Fe–Bi sites well-suited for the multistep sulfur reduction sequence and promoted rate performance.

By contrast, the Zn–Bi motif offers a complementary profile. Zn (3d^10^) is nominally redox-inactive under operating conditions, but exhibits borderline-soft Lewis acidity and robust coordination stability. Coupling Zn with Bi induces ligand-field perturbations and local structural distortion around Zn via interaction of Bi 6p with Zn’s filled 3d shell. Although Zn alone shows little density of states near E_F_, the presence of Bi polarizes and redistributes charge, activating valence electron density (Figures S23–S25).

From the CN, ZnBi@CN, and FeBi@CN models (Figure 1a), a decrease in the energy gap from 3.52 eV for CN to 0.48 eV for ZnBi@CN and 0.25 eV for FeBi@CN (Figure 4a) was observed. The elevated highest occupied molecular orbital (HOMO) level promotes electron donation from the catalyst to sulfur species during reduction, while the lowered lowest unoccupied molecular orbital (LUMO) level facilitates electron acceptance from sulfur species during oxidation in FeBi@CN and ZnBi@CN.

**Figure 4:**
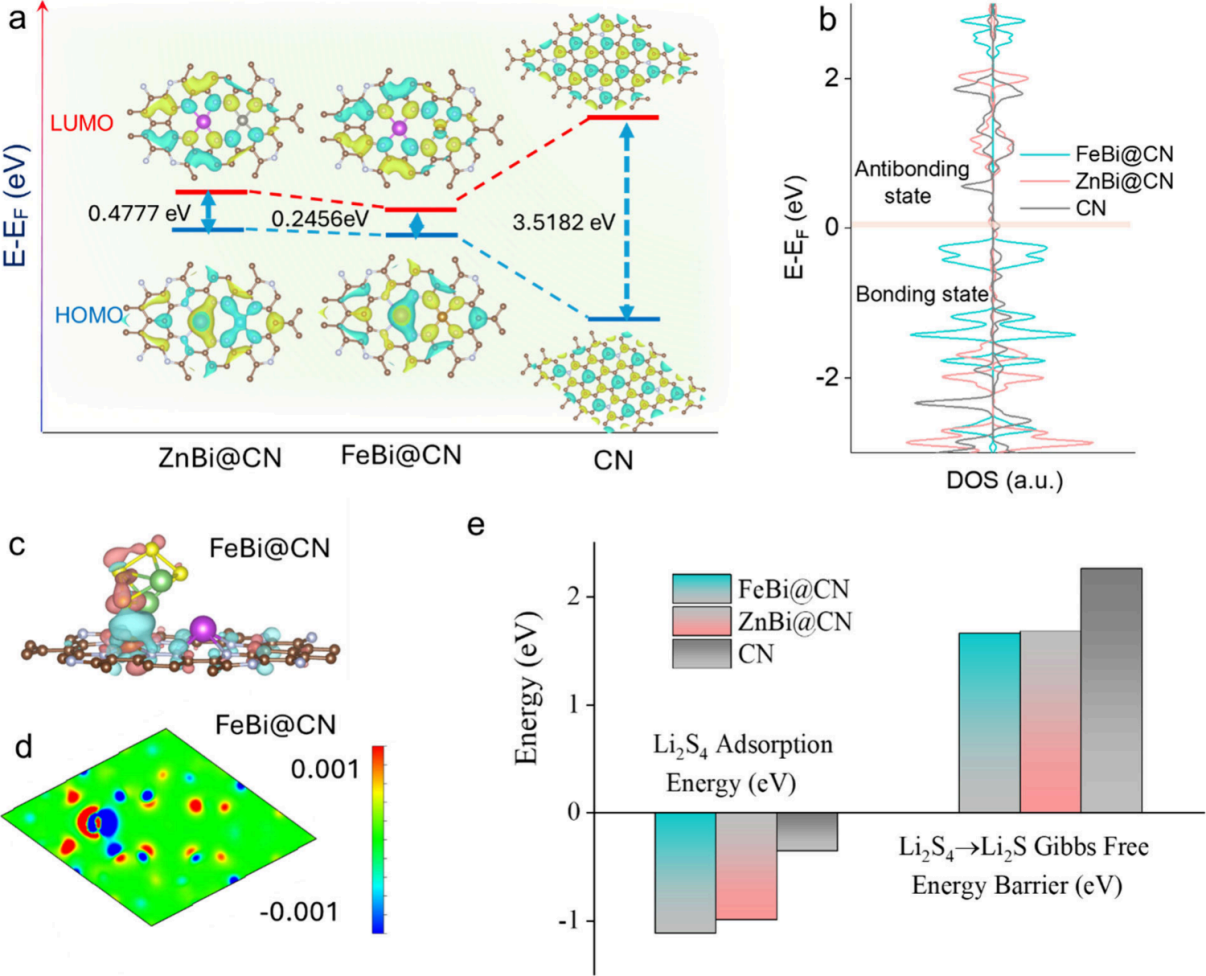
(a) Schematic representation of the HOMO and LUMO for FeBi@CN, ZnBi@CN, and CN. (b) Projected DOS for bonding state orbital hybridization of Li_2_S_4_ on FeBi@CN, ZnBi@CN, and CN. (c) Side view of the charge-density difference plots for FeBi@CN interacting with Li_2_S_4_. Red and blue isosurfaces represent electron accumulation and electron depletion, respectively, with an isosurface cutoff of 0.001 e^–^/bohr^3^. (d) 2D projection of the charge density contour of FeBi@CN-Li_2_S_4_ at the α section as seen in (c). (e) Adsorption energy of Li_2_S_4_ and the Gibbs free energy change for the conversion of Li_2_S_4_ to Li_2_S on the surfaces of FeBi@CN, ZnBi@CN, and CN catalysts.

Figure 4b illustrates the energy levels corresponding to the orbital hybridization between Li_2_S_4_ and the ZnBi@CN, FeBi@CN, and CN surfaces. The calculated density of states (DOS) for polysulfide on these catalyst surfaces shows distinct variations in the bonding states around the sulfur Fermi level. Specifically, the FeBi@CN surface exhibits the highest electron density near the Fermi level, followed by ZnBi@CN. In the Fe–Bi motif, the partially filled Fe 3d orbitals and accessible redox states enable S–S bond activation and d-orbital-mediated electron transfer, causing polysulfide adsorption to shift bonding states toward the Fermi level and thereby accelerate charge transfer to sulfur species (Figure S26).

To further investigate the electron transfer dynamics between polysulfides and the different catalysts, differential charge density analysis was performed, revealing distinct regions of electron depletion (blue) around the catalyst surface, and electron accumulation (red) at the Li_2_S_4_ anchoring sites. The electron depletion observed around the catalyst surface suggests a localized reduction in electron density, likely driven by the interaction between the catalyst and the polysulfide species. This electron withdrawal could induce a polarization of the polysulfide intermediates, facilitating their charge redistribution, a critical step for the redox reactions involved in lithium–sulfur battery operation. In contrast, the electron accumulation at the Li_2_S_4_ anchoring sites indicates a significant localized increase in electron density. This suggests that the catalyst plays a key role in stabilizing the polysulfide intermediates, particularly the Li_2_S_4_ species, through favorable electronic interactions. The enhanced electron density at these sites likely lowers the activation energy for the reduction or oxidation processes, promoting more efficient electron transfer between the polysulfides and the catalyst surface. As a result, the catalyst not only facilitates the stable anchoring of the polysulfides but also accelerates their transformation, which is crucial for improving the cycling stability and electrochemical performance of lithium–sulfur batteries. Importantly, this accumulation around the anchoring sites can lower the energy barrier for polysulfide transformation, promoting faster and more efficient electrochemical reactions. The 2D cross-sectional maps, particularly the α section of Figure 4c, provide a clear visualization of the electron distribution. The pronounced electron accumulation around the Fe atoms is particularly notable, suggesting that the Fe-based catalyst facilitates stronger electronic modulation compared to other catalysts under study. This enhanced electronic interaction likely plays a pivotal role in improving the catalytic activity of Fe-based catalysts, as it enhances the adsorption and transformation of polysulfides, ultimately boosting the performance of lithium–sulfur batteries. Notably, robust coupling forms between Bi 6p and Fe 3d orbitals: Bi acts as a soft Lewis-acid center with 6p sites that strengthen π-type polysulfide adsorption (Figure 1a), while Fe contributes d-orbital electrons to accelerate charge transfer. This p–d hybridization generates a polarized bimetallic environment that synergistically facilitates S–S bond cleavage, accelerates Li insertion, and overall promotes polysulfide redox conversion.

For the critical Li_2_S_4_ → Li_2_S conversion, which accounts for two-thirds of the theoretical capacity in Li–S batteries, the Gibbs free energy barriers of ZnBi@CN (1.66 eV) and FeBi@CN (1.68 eV) are nearly identical and substantially lower than that of CN (2.26 eV) (Figures 4e and S27). In contrast, clear differences emerge in Li_2_S_4_ adsorption: CN exhibits the weakest interaction (−0.35 eV), whereas DAC incorporation greatly strengthens adsorption, with FeBi@CN and ZnBi@CN reaching – 1.12 eV and – 0.99 eV, respectively (Figure 4g). The corresponding adsorption configurations of S_8_, Li_2_S_8_, Li_2_S_6_, Li_2_S_4_, Li_2_S_2_, Li_2_S are shown in Figures S28–S31. Computed adsorption energies for Li_2_S_4_ place FeBi@CN near the optimal binding window for efficient polysulfide conversion, consistent with the relatively high capacities and Q_2_/Q_1_ ratios (Figure S16).

In summary, DFT calculations show that both Fe–Bi and Zn–Bi create multifunctional dual-atom sites with high atom utilization and tunable behavior. In Fe–Bi, electronic reconstruction, spin regulation, and interfacial coupling enhance redox kinetics by shifting the Fe 3d states toward the Fermi level, thereby facilitating charge transfer, S–S bond cleavage, and multistep Li insertion. In contrast, ZnBi, though less electronically activating, strongly stabilizes LiPS intermediates and promotes dense Li_2_S nucleation, in line with potentiostatic deposition tests and its high cycling stability. Collectively, these results support a complementary bifunctional mechanism: Fe in FeBi@CN primarily accelerates electron-transfer–limited steps, while Zn in ZnBi@CN enhances intermediate stabilization and mitigates shuttle effects, yielding fast kinetics for FeBi@CN and outstanding durability despite the lower catalytic activity for ZnBi@CN.

In conclusion, our results demonstrate that asymmetric FeBi@CN and ZnBi@CN dual-atom sites accelerate sulfur redox kinetics, lower the Li_2_S nucleation barrier, and suppress parasitic reactions, thereby enabling both high-rate capability and long-term stability in Li–S cells. The DAC architecture effectively mitigates polysulfide shuttling and sustains catalytic activity over extended cycling, with FeBi@CN retaining 76% of its initial capacity and ZnBi@CN maintaining 81% after 1000 cycles, underscoring the robustness of both designs. Mechanistically, ligand-field coupling between Bi single atoms and 3d centers regulates the local electronic state density, spin configuration, and structural distortion at the active sites, accounting for the experimentally observed high activity of Fe–Bi. More broadly, atomically engineered Fe–Bi and Zn–Bi motifs, built from earth-abundant elements, offer a viable pathway toward efficient, durable, and sustainable Li–S batteries, while establishing a transferable design principle for p-block/3d bimetallic catalysts in related electrochemical systems.

## Supplementary Material



## Notes

Synthesis details, calculations, and additional figures (PDF)
