# Supplementary material for: Engineering p–d Coupling at Fe–Bi and Zn–Bi Sites for Efficient Li–S Conversion
Source: Nano Lett. 2025 Nov 5;25(46):16427–34. doi: 10.1021/acs.nanolett.5c04356 (PMC12636070; doi:10.1021/acs.nanolett.5c04356)
Supplement: Supplementary file 1 [file nl5c04356_si_001.pdf]

Engineering p–d Coupling at Fe-Bi and Zn-Bi Sites for Efficient Li–S Conversion

Jing Yu[1,2], Zhifu Liang[3], Xianggui Zhou[4], Chaoqi Zhang[5], Ivan Pinto-Huguet[2], Oleg Usoltsev[6], Chaoyue Zhang[7,], Alex W. Robertson[8], Andreu Cabot,[1,9,*] Jordi Arbiol[2,9,*]*

1 Catalonia Institute for Energy Research (IREC), Sant Adrià de Besòs, Barcelona 08930, Catalonia, Spain

2 Catalan Institute of Nanoscience and Nanotechnology (ICN2), CSIC and BIST, Campus UAB, Bellaterra, 08193 Barcelona, Catalonia, Spain

3 College of Ocean Science and Engineering, Shanghai Maritime University, 201306 Shanghai, China

4. South China Advanced Institute for Soft Matter Science and Technology, School of Emergent Soft Matter, South China University of Technology, Guangzhou 510640, China

5 College of Materials Science and Engineering, Fuzhou University, No.2, Xueyuan Road, Minhou County, Fuzhou City, Fujian Province 350108, China

6 CELLS-ALBA Synchrotron, Carrer de la Llum, 2, 26, 08290 Cerdanyola del Vallès, Barcelona, Catalonia, Spain

7 Department of Chemistry and Biochemistry, University of California, Los Angeles, CA, USA

8 Department of Physics, University of Warwick, Coventry, CV4 7AL, UK

9 ICREA Pg. Lluis Companys, Barcelona 08010, Catalonia, Spain

*Corresponding authors: *C.Y.Z.* Chyzhang0810@ucla.edu; A.C. acabot@irec.cat; J.A. arbiol@icrea.cat

Experimental Section

Synthesis of FeBi@CN and ZnBi@CN: Chloroanilic acid (417.96 mg, 2 mmol) was added to a three-necked round-bottom flask under an argon atmosphere and cooled in an ice bath. N-methyl-2-pyrrolidone (NMP, 12 mL) was used as solvent, into which melamine (504.48 mg, 4 mmol), $BiCl_3$ (20 mg), and an equimolar amount of the selected transition metal chloride ($FeCl_2$, $ZnCl_2$) were introduced at ~0 °C. While stirring vigorously, several drops of concentrated H_2SO_4 were added slowly. After 20 min of continuous stirring, the ice bath was removed, and the mixture was warmed to room temperature before being heated to 170 °C for 24 h under argon. The product was cooled to room temperature, vacuum-filtered, washed sequentially with ethanol and water (×3), and freeze-dried for 24 h. The obtained black solid was annealed at 700 °C for 3 h under argon with a heating ramp of 5 °C min^{-1}.

Preparation of FeBi@CN/S and ZnBi@CN/S: Composites were prepared in three steps: (i) TM-Bi@CN and sulfur powder were mixed in a 1:3 mass ratio, (ii) the mixture was ground thoroughly using a mortar, and (iii) the ground product was heat-treated at 155 °C for 12 h to yield FeBi@CN/S and ZnBi@CN/S.

LSB assembly: CR2032-type coin cells were fabricated in an argon-filled glove box. Cathodes were prepared by mixing TM-Bi@CN/S, Super P, and PVDF (8:1:1 by weight), casting onto Al foil, and drying under low vacuum at 80 °C overnight. S loading was set at ~1.2 mg cm^{-2}. Disks of 12 mm diameter were punched from the dried film. The electrolyte (40 μL) consisted of 1.0 M LiTFSI and 0.2 M $LiNO_3$ in DOL/DME (1:1 v/v). Lithium foil was used as the anode.

Synthesis of Li_2S_6 solution: A Li_2S_6 solution was obtained by dissolving elemental S and Li_2S with the molar ratio of 5:1 in a DME/DOL mixture and heating at 80 °C overnight.

Symmetric cell assembly: Symmetric cells were prepared using electrodes composed of FeBi@CN and ZnBi@CN, Super P, and PVDF (8:1:1 by weight). Two identical electrodes (~0.5 mg cm^{-2} loading) served as both working and counter electrodes. The electrolyte (40 μL) contained 0.5 M Li_2S_6 and 1.0 M LiTFSI in DOL/DME (1:1 v/v). Cyclic voltammetry was performed within a potential window of –1.5 V to 1.5 V, corresponding to the potential difference between the two identical electrodes, centered at their equilibrium point ($\Delta E = 0$).

Li_2S nucleation experiment: Coin cells were assembled with TM-Bi@CN as the working electrode, lithium foil as the counter electrode, Celgard 2400 as the separator, and 0.5 mM Li_2S_6 solution as electrolyte. Cells were first discharged to 2.06 V at 2.56 mA cm^{-2}, followed by potentiostatic deposition at 2.05 V.

Material characterization: Atomic-resolution imaging was performed on a Thermo Fisher Spectra 300 double aberration-corrected STEM at 300 kV with a Super-X EDS detector. Inductively coupled plasma optical emission spectrometry (ICP-OES) Analysis. To accurately determine the bulk elemental composition of the FeBi@CN and ZnBi@CN catalysts, ICP-OES measurements were performed. Solid catalyst samples (2 mg) were first digested in 2 mL of aqua regia to form a stock solution. A 0.1 mL aliquot of this solution was then filtered and diluted with 9.9 mL of 2% HNO_3 to prepare the measurement solution. Elemental concentrations of Fe, Zn, and Bi were measured in the diluted solutions by ICP-OES, and the results were back-calculated using the known dilution factor to determine the elemental content in the original solid samples.

X-ray Absorption Spectroscopy (XAS): XAS experiments were performed at the CLAESS beamline (ALBA synchrotron). Ex situ spectra were processed using the DEMETER software package (Athena/Artemis), including calibration, normalization, and EXAFS fitting with FEFF-based models.

DFT calculation: First-principles calculations were performed using DFT in the Vienna Ab initio simulation package (VASP). The electron exchange and correlation effects were treated using the generalized gradient approximation (GGA) in the Perdew-Burke-Ernzerhof (PBE) pseudopotential parameterization. The projector augmented wave (PAW) method was employed with a plane wave cutoff energy of 500 eV. Doping effects were simulated using a 2×2×1 TM-Bi/CN supercell. A vacuum slab of 16 Å was included to prevent interlayer interactions. Structural relaxation was performed using a 2×2×1 gamma-centered k-point mesh with a cutoff energy of 500 eV. The convergence criteria for energy and force were set to 1×10^{-5} eV and 0.05 eV/Å, respectively. The adsorption energy (ΔEad) was calculated as follows:

$$\Delta E_{ad} = E_{\mathrm{surf+ad}} - E_{\mathrm{surf}} - E_{\mathrm{ad}}$$

where $E_{\mathrm{surf+ad}}$ is the energy of LiPS adsorbed on the surface, E_{surf} is the energy of the clean surface, and E_{ad} is the energy of free LiPS. In addition, the differential spin density was calculated to

examine the spin electron transfer during the adsorption process. The differential electron density ($\Delta\rho$) was determined using the following formula:

$$\Delta\rho = \rho_{AB} - \rho_{A} - \rho_{B}$$

where ρ_{AB} is the spin density of the system after adsorption, and ρ_{A} and ρ_{B} are the spin densities of the adsorbed species and catalyst, respectively.

The calculation formula of Gibbs free energy is:

$$\Delta G(S_8\text{-}Li_2S_8) = E_{Li_2S_8} - E_{S_8} - 2E_{Li^+}$$

$$\Delta G(Li_2S_8\text{-}Li_2S_6) = E_{Li_2S_6} - E_{Li_2S_8} + 0.25E_{S_8}$$

$$\Delta G\ (Li_2S_6\text{-}Li_2S_4) = E_{Li_2S_4} - E_{Li_2S_6} + 0.25E_{S_8}$$

$$\Delta G(Li_2S_4\text{-}Li_2S_2) = E_{Li_2S_2} - E_{Li_2S_4} + 0.25E_{S_8}$$

$$\Delta G(Li_2S_2\text{-}Li_2S) = E_{Li_2S} - E_{Li_2S_2} + 0.125E_{S_8}$$

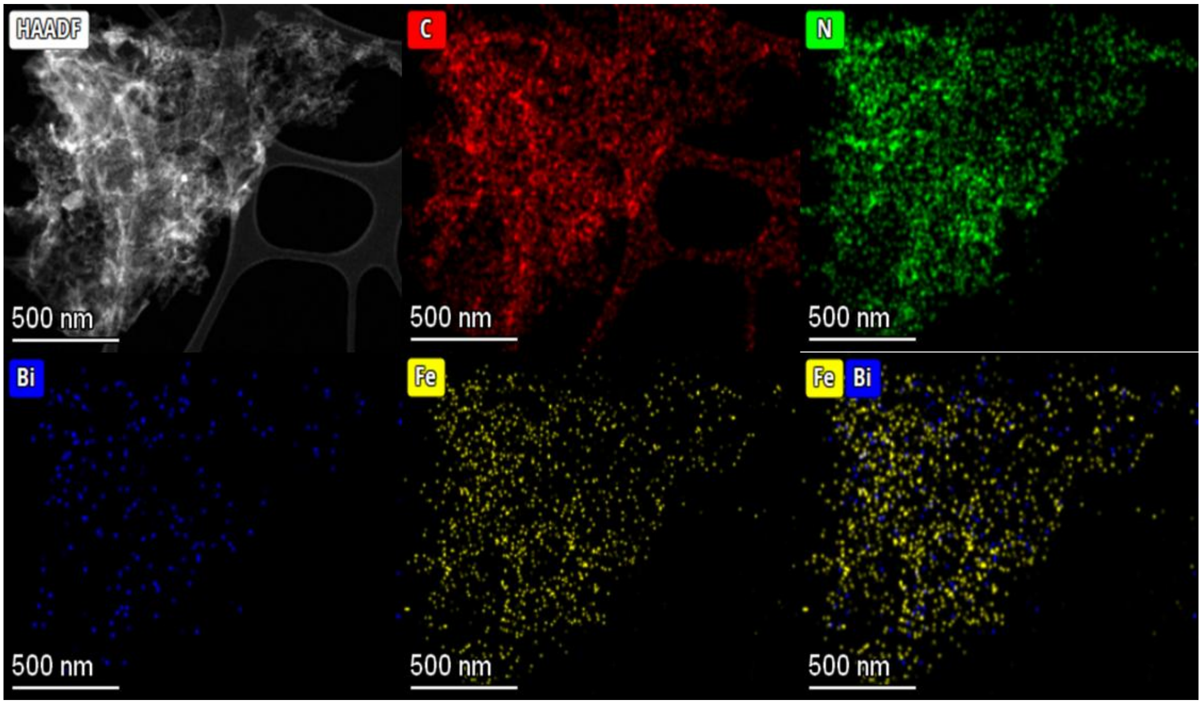

Figure S1. STEM-HAADF image and corresponding EDS elemental maps of FeBi@CN.

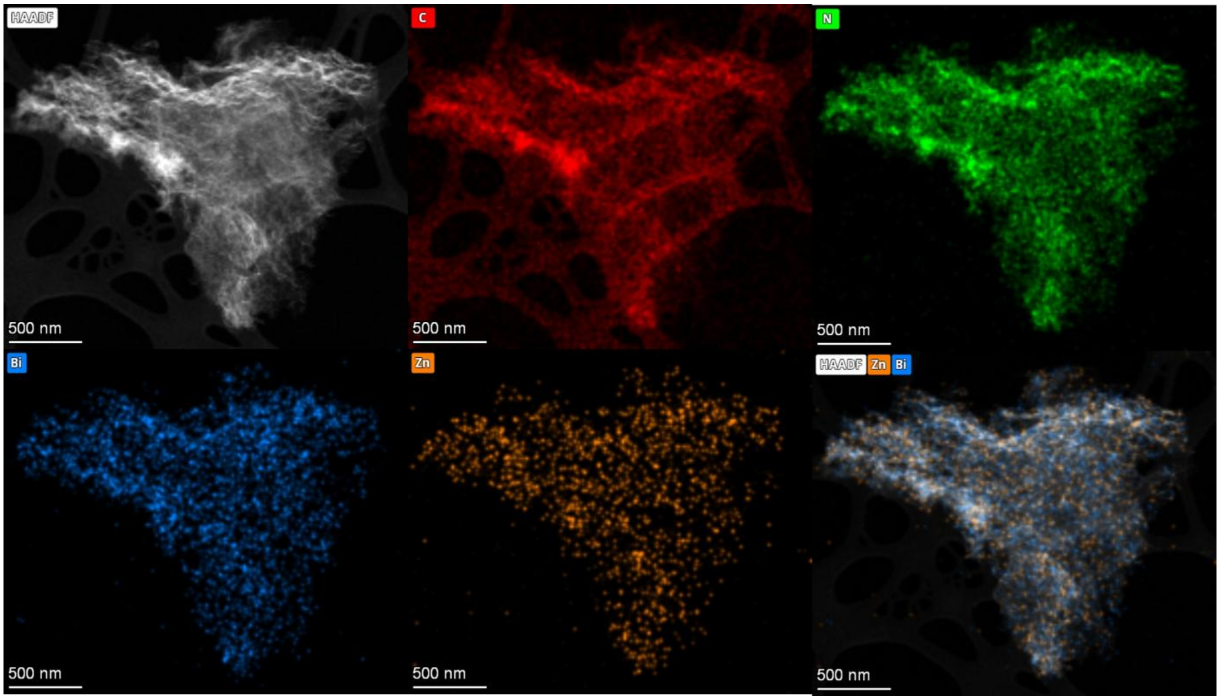

Figure S2. STEM-HAADF image and corresponding EDS elemental maps of ZnBi@CN.

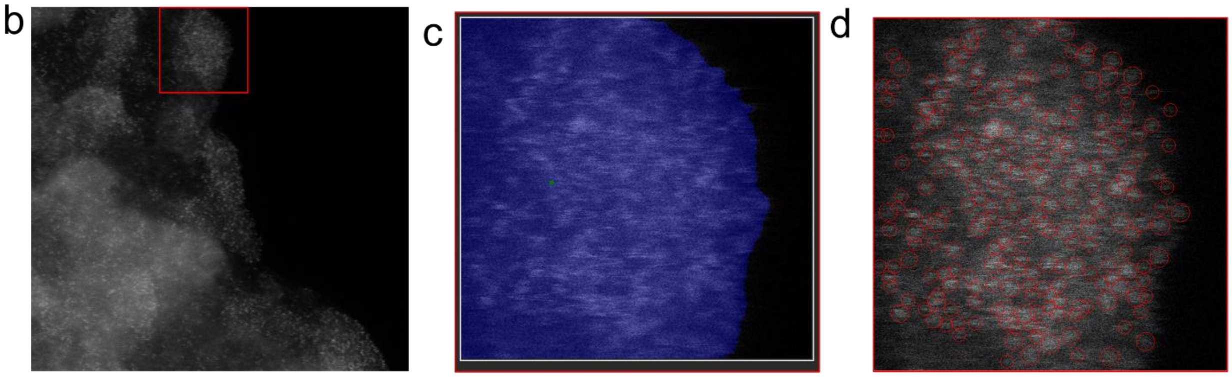

Figure S3. STEM Atom counting methodology, including segmentation and counted atoms.

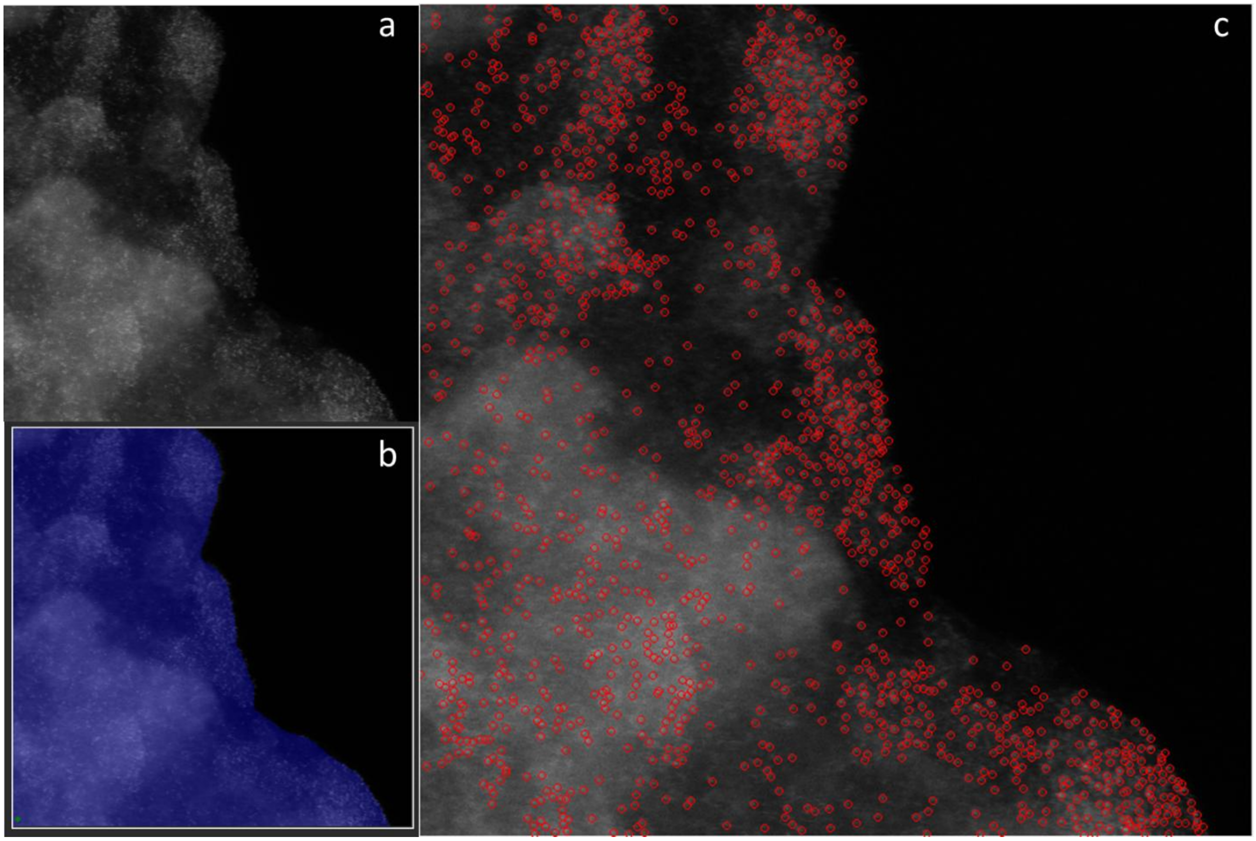

Figure S4. AC HAADF STEM images of FeBi@CN. Atom counting = 7 atoms nm^{-2}.

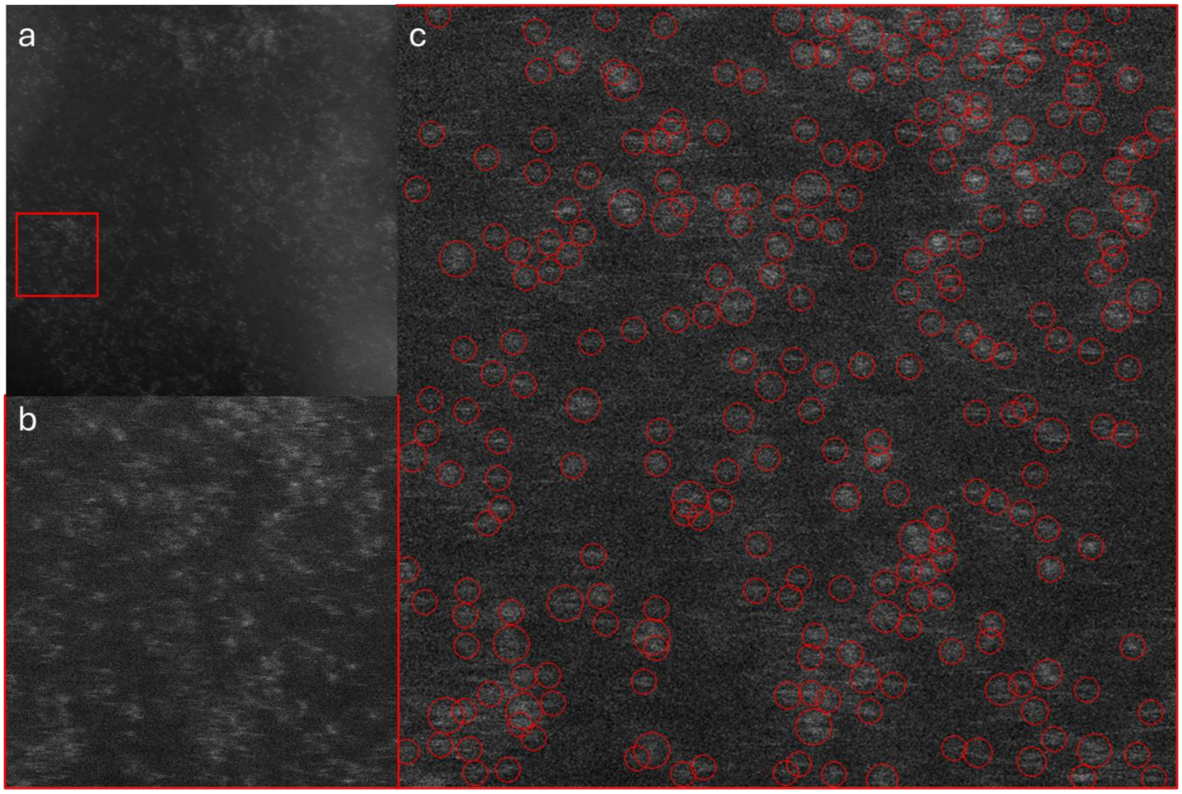

Figure S5. AC HAADF STEM images of ZnBi@CN marking atomic pairs. Atom counting = 7 atoms nm^{-2}

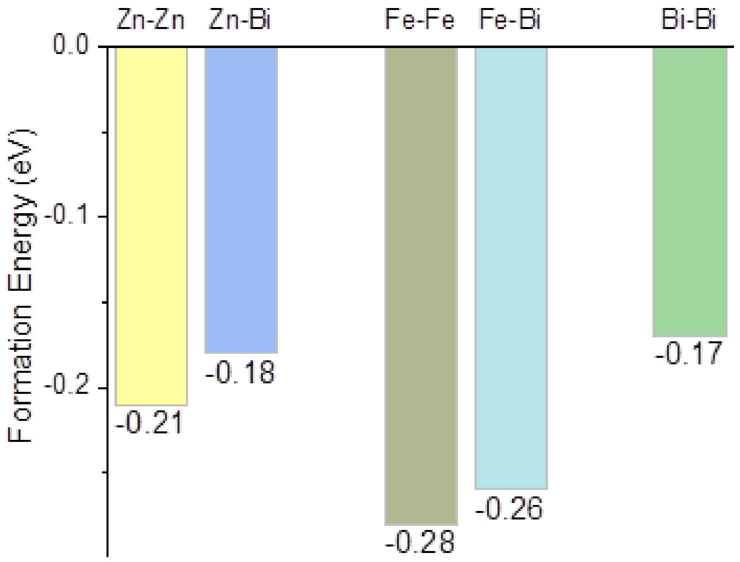

Figure S6. DAC formation energies for ZnZn@CN, ZnBi@CN, FeFe@CN, FeBi@CN, and BiBi@CN.

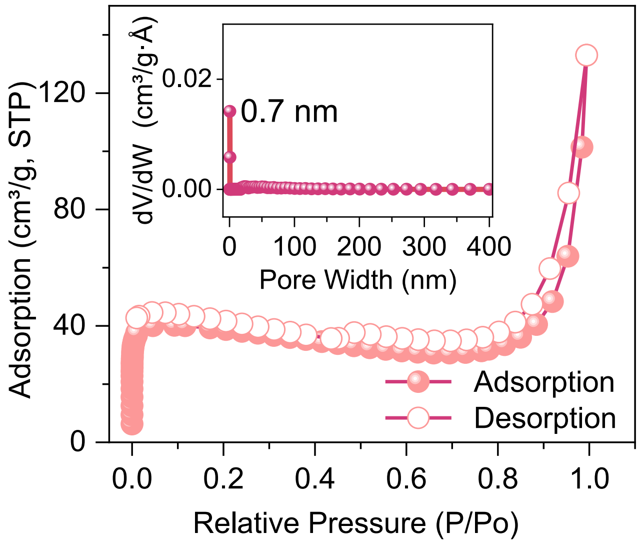

Figure S7. Nitrogen adsorption–desorption isotherms of CN and corresponding pore size distribution.

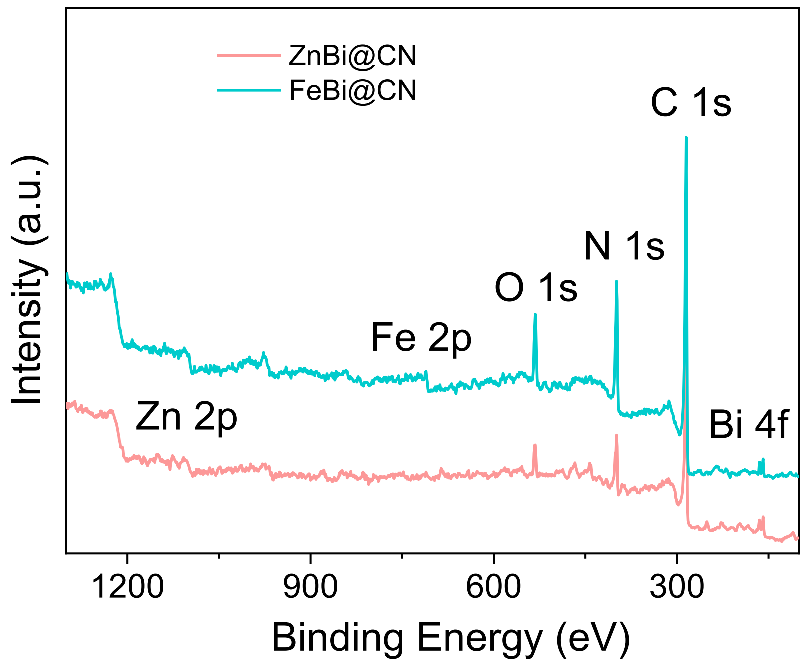

Figure S8. Survey XPS spectrum of the FeBi@CN and ZnBi@CN samples.

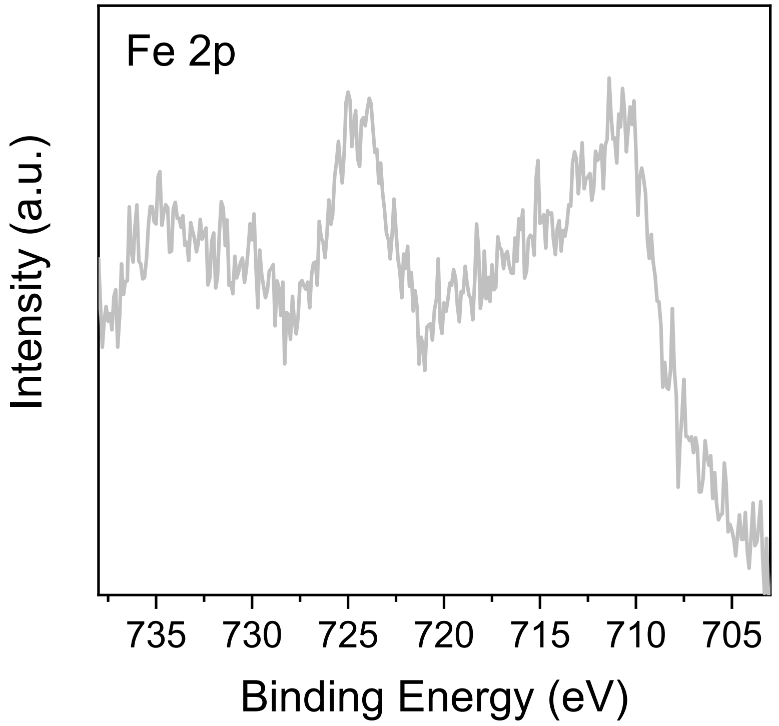

Figure S9. High-resolution Fe 2p XPS spectrum of FeBi@CN.

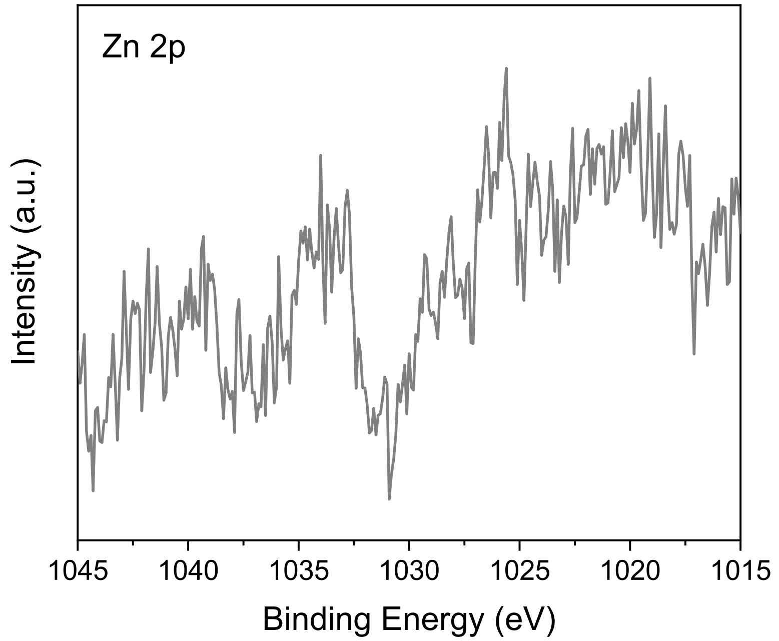

Figure S10. High-resolution Zn 2p XPS spectrum of ZnBi@CN.

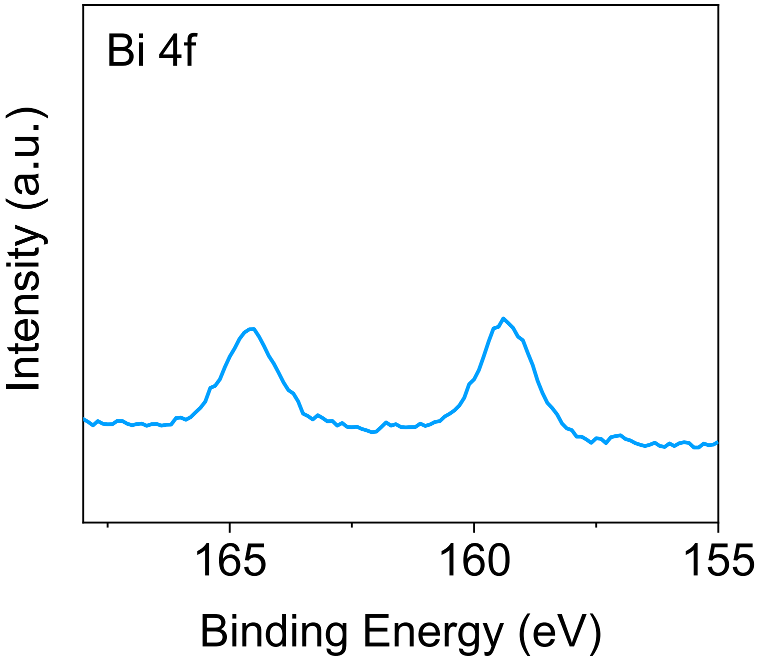

Figure S11. High-resolution Bi 4f XPS spectrum of ZnBi@CN.

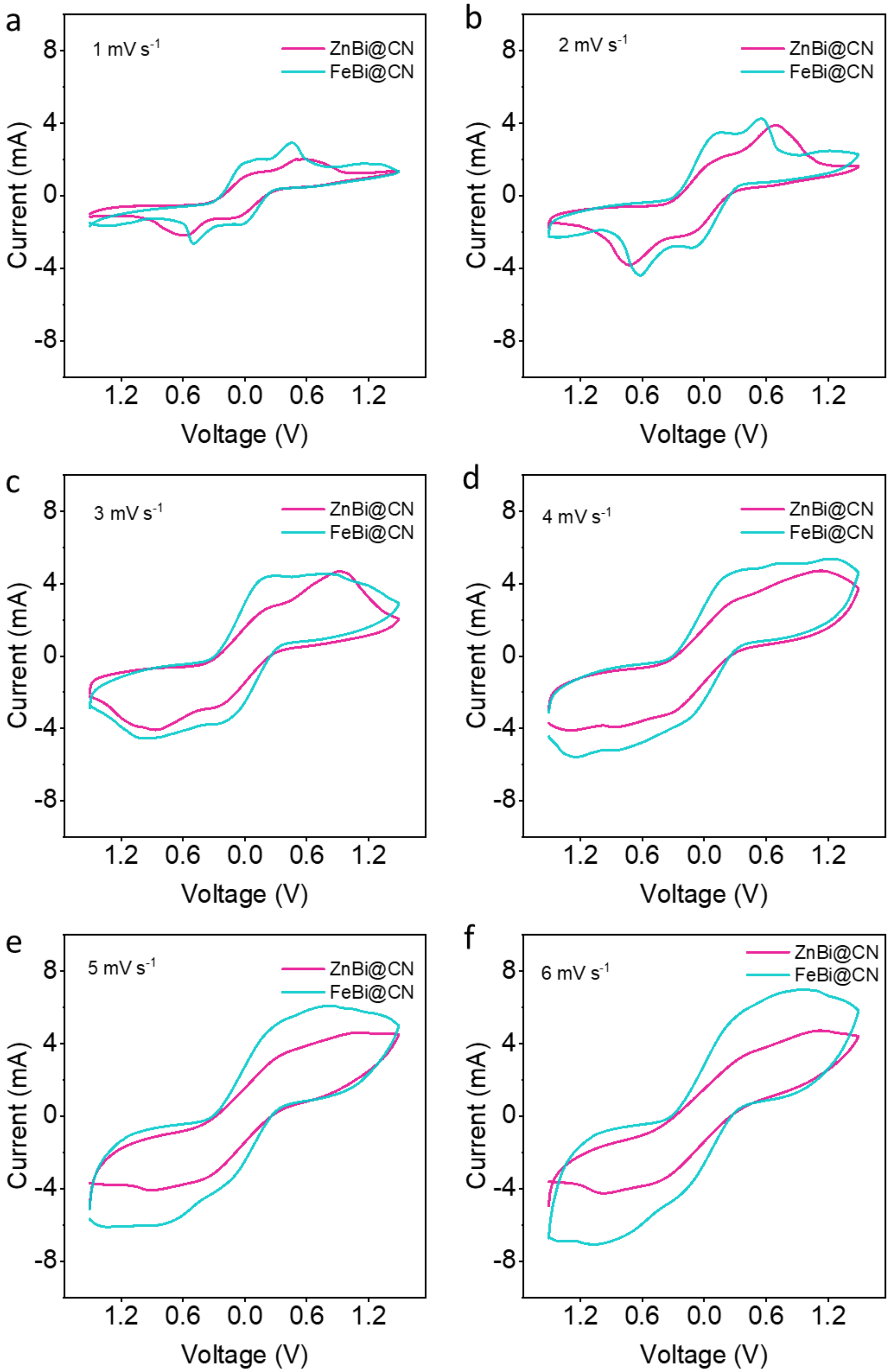

Figure S12. CV curves obtained from FeBi@CN and ZnBi@CN symmetric cells at different scan

speed across 1-6 mV/s.

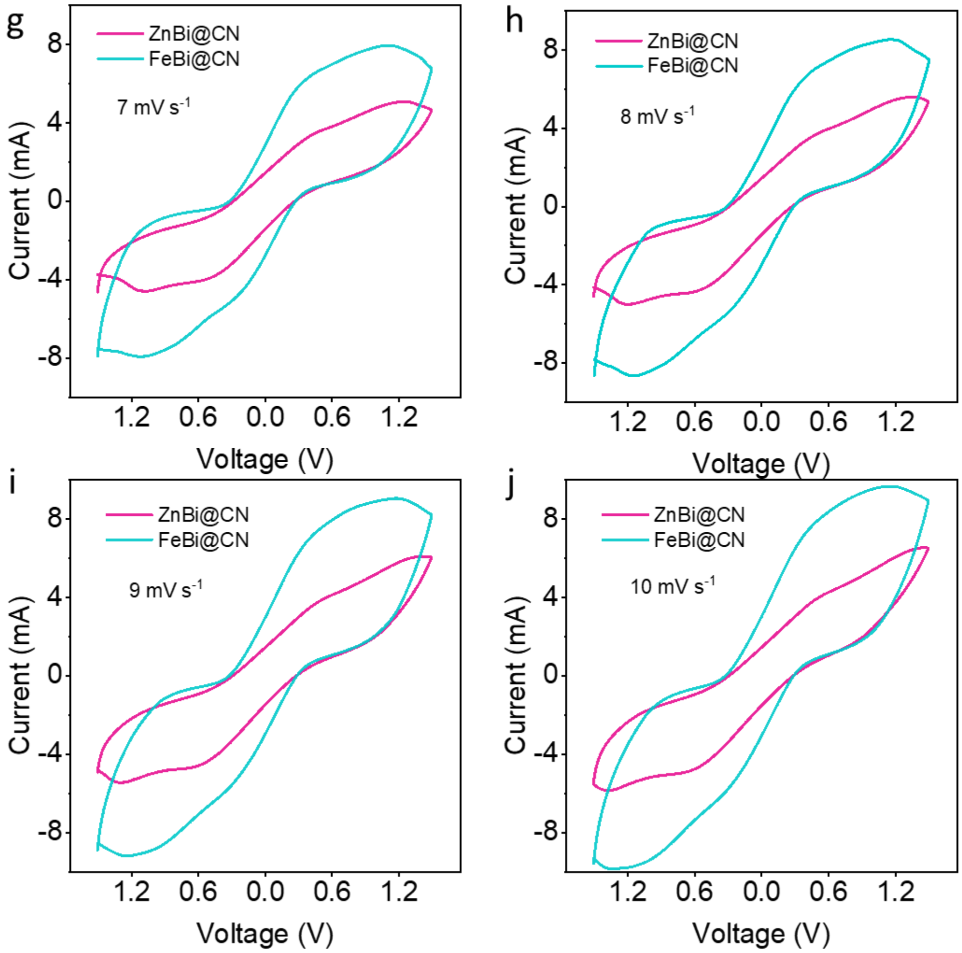

Figure S13. CV curves obtained from FeBi@CN and ZnBi@CN symmetric cells at different scan speed across 7-10 mV/s

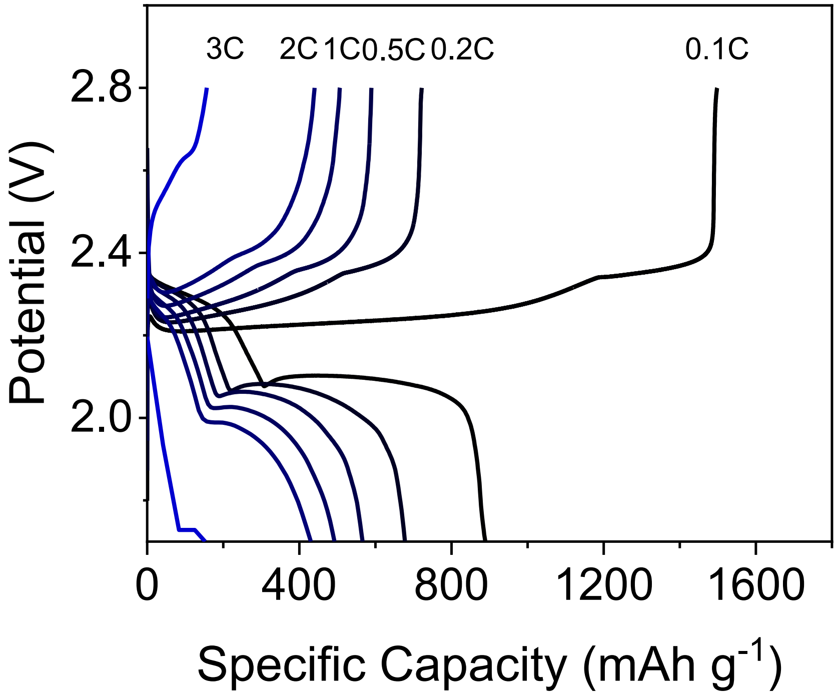

Figure S14. GCD curves of a SP/S cell at different rates.

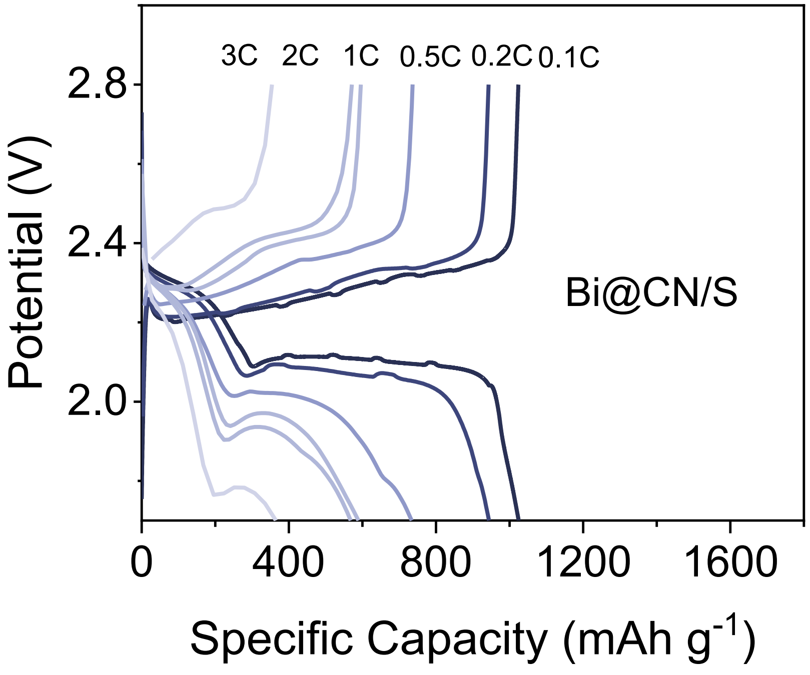

Figure S15. GCD curves of a Bi@CN/S cell at different rates.

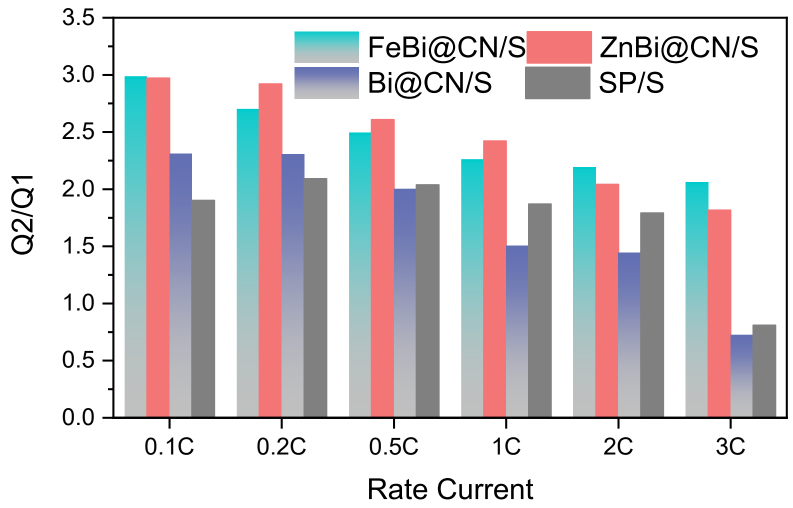

Figure S16. Q_2/Q_1 values of FeBi@CN/S, ZnBi@CN/S, and SP/S.

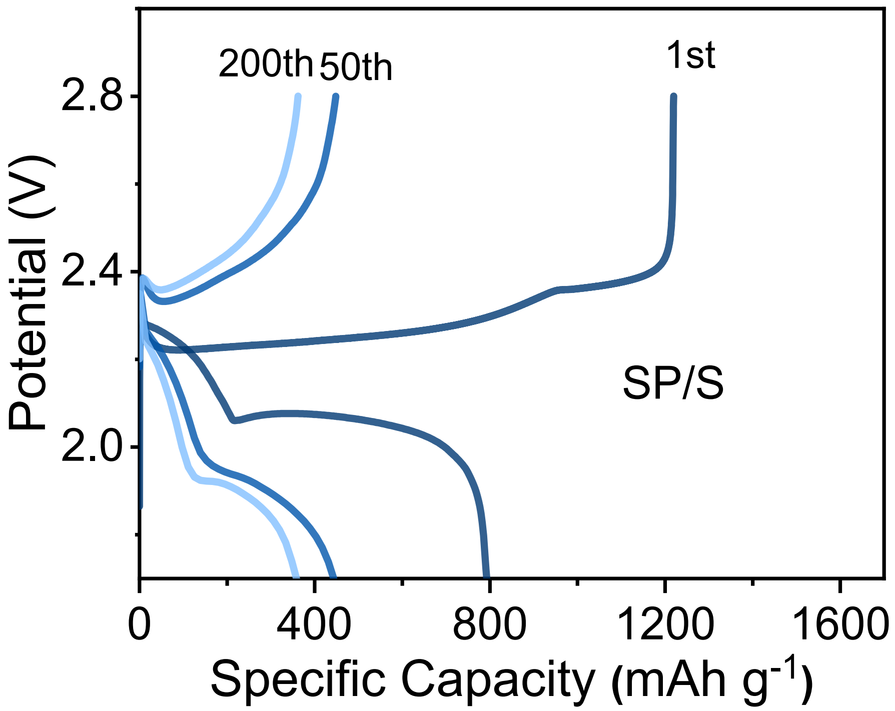

Figure S17. GCD curves of a SP/S cell before, during, and after 200 cycles.

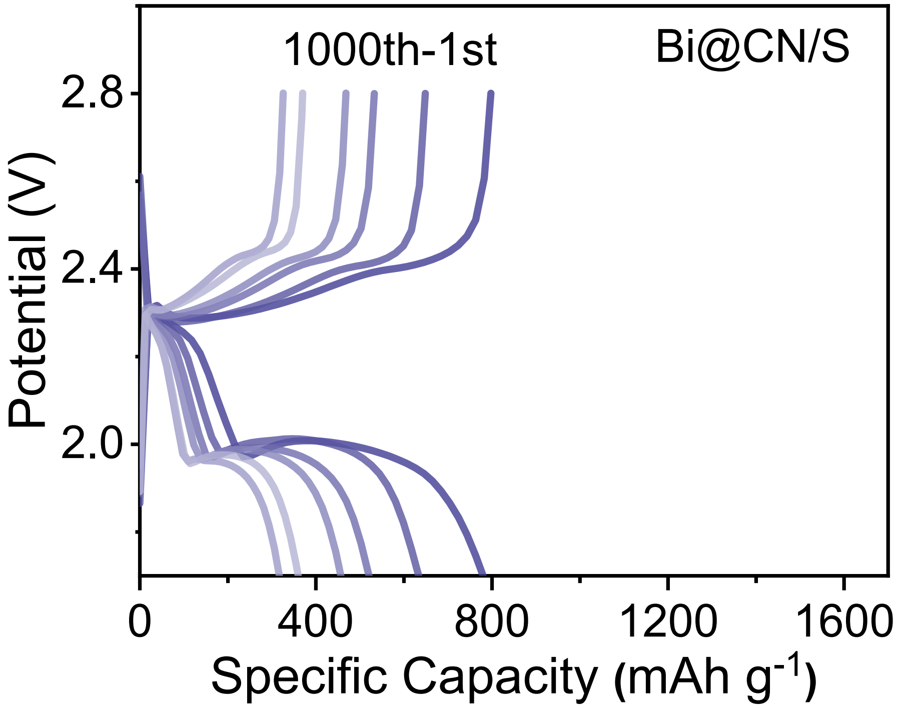

Figure S18. GCD curves of a Bi@CN/S cell before, during, and after 1000 cycles.

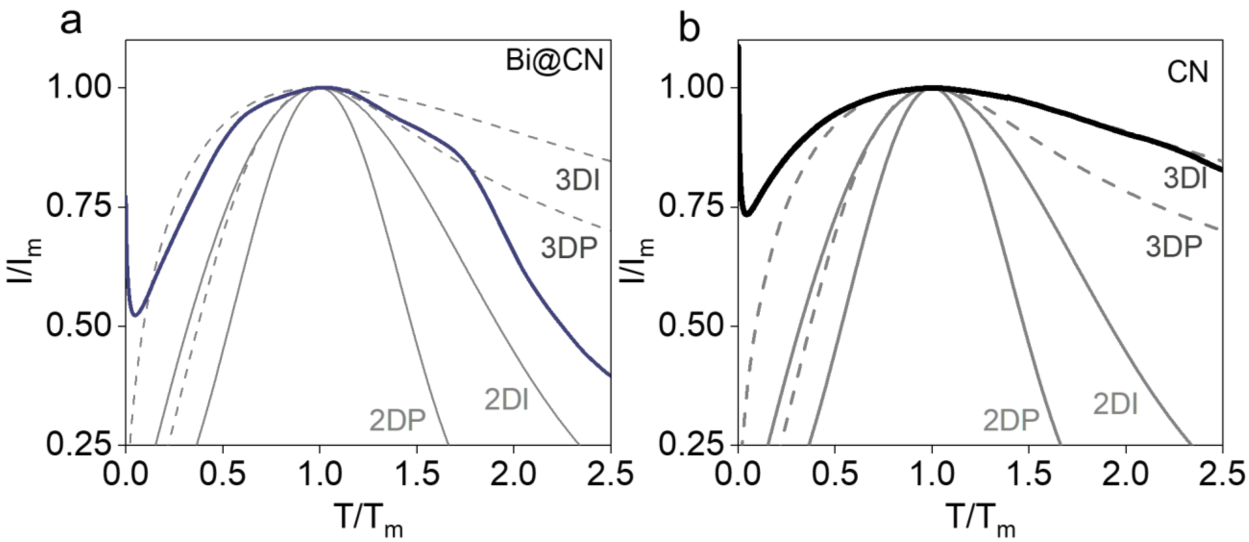

Figure S19. Normalized t/t_m and $(I/I_m)^2$ plots (solid lines) compared with theoretical 3D Instantaneous, 3D Progressive, 2D Instantaneous, and 2D Progressive nucleation models for Bi@CN and CN electrodes.

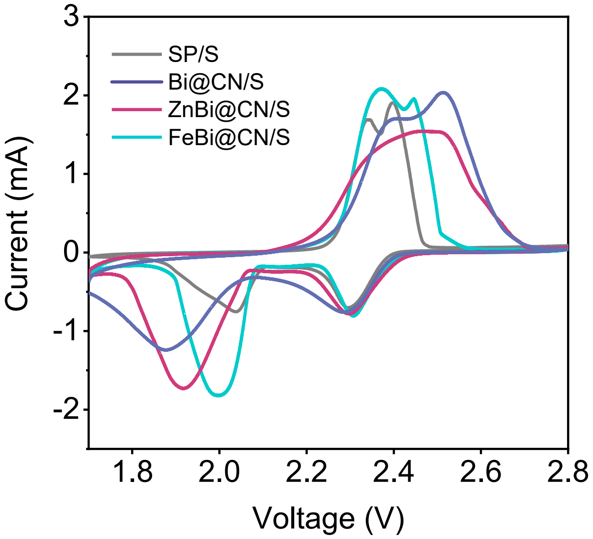

Figure S20. CV curves of SP/S, Bi@CN/S, ZnBi@CN/S, FeBi@CN/S at the scan speed of 3 mV s^{-1}.

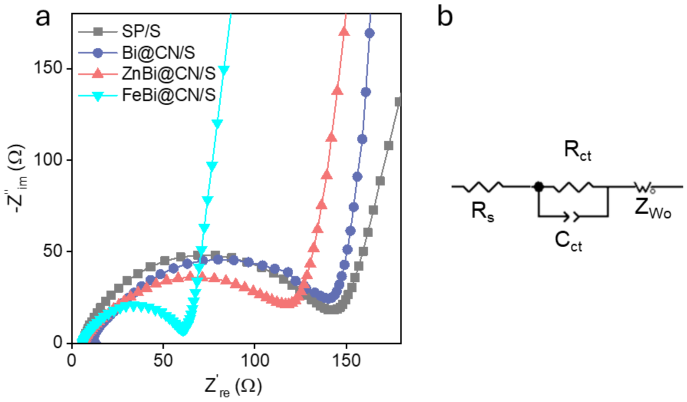

Figure S21. Electrochemical impedance spectroscopy (EIS) results of SP/S, Bi@CN/S, ZnBi@CN/S, FeBi@CN/S and it's corresponding model circuit .

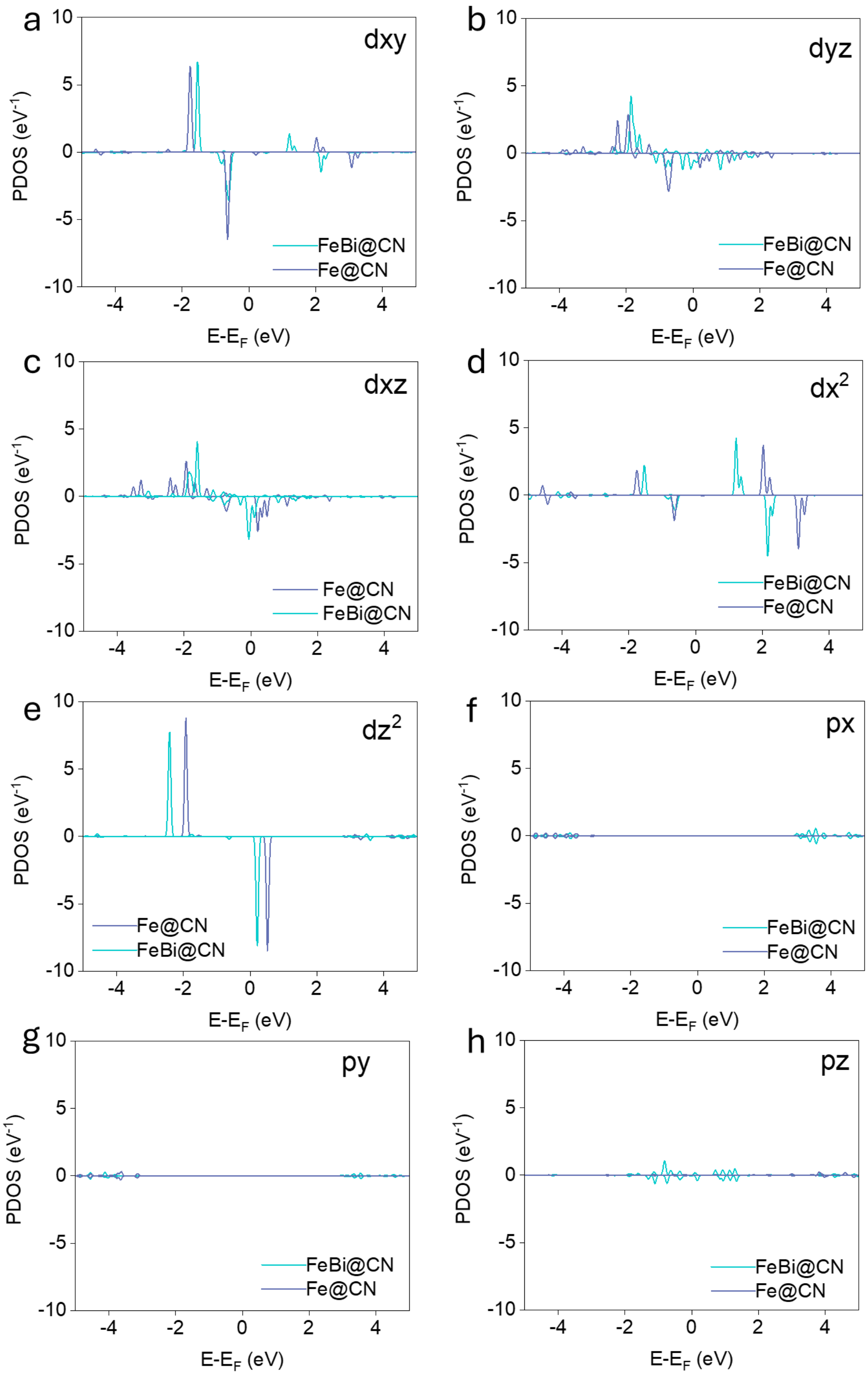

Figure S22. PDOS of d orbitals (d_{xy}, d_{yz}, d_{z}^{2}, d_{xz}, and d_{x}^{2}) and p orbitals (p_x, p_y, and p_z) of Fe in FeBi@CN and Fe@CN.

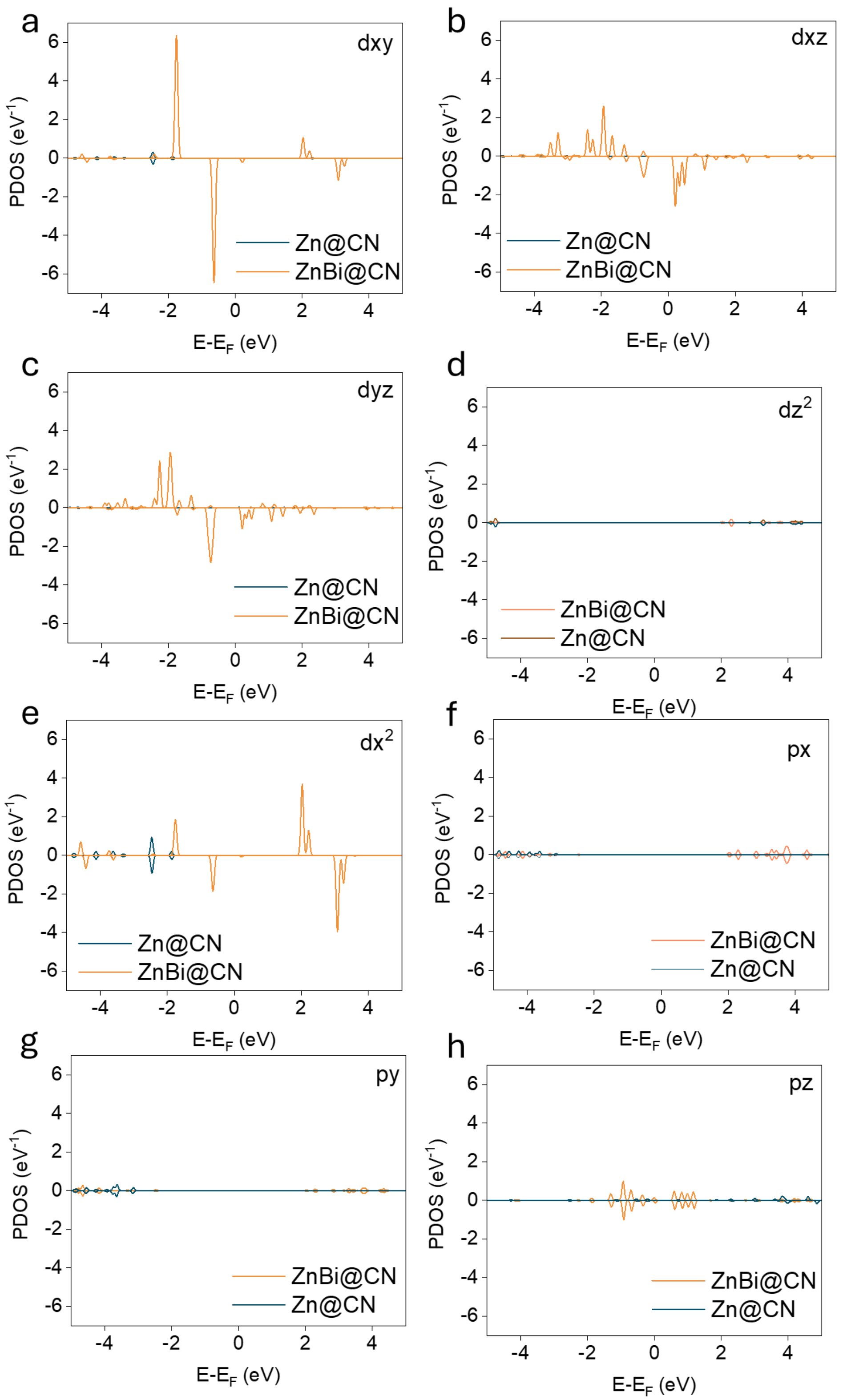

Figure S23. PDOS of d orbitals (d_{xy}, d_{yz}, d_{z}^{2}, d_{xz}, and d_{x}^{2}) and p orbitals (p_x, p_y, and p_z) of Zn in ZnBi@CN and Zn@CN.

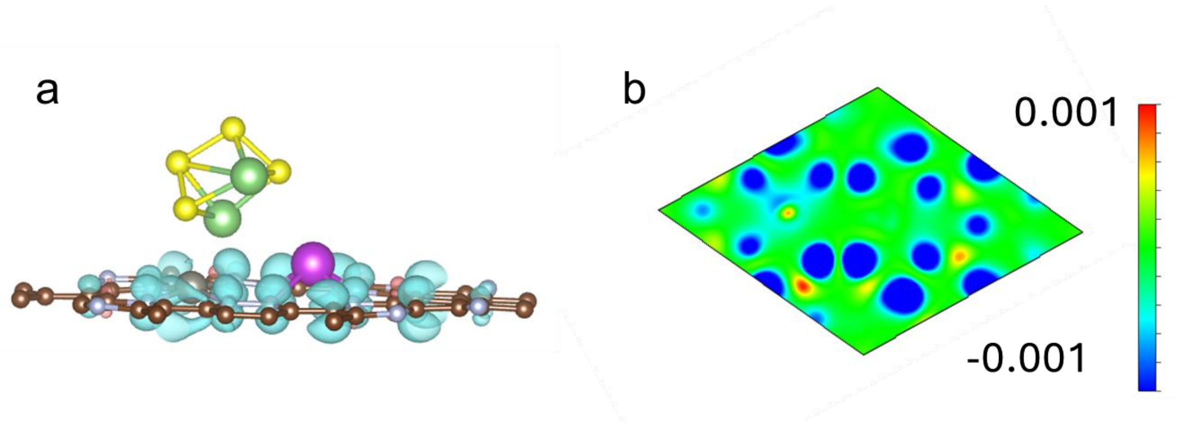

Figure S24. (a) Side view of the charge-density difference plots for ZnBi@CN interacting with Li_2S_4. Red and blue isosurfaces represent electron accumulation and electron depletion, respectively, with an isosurface cutoff of 0.001 $e^-/Bohr^3$. (b) 2D projection of the charge density contour of ZnBi@CN-Li_2S_4 at the α section as seen in (a).

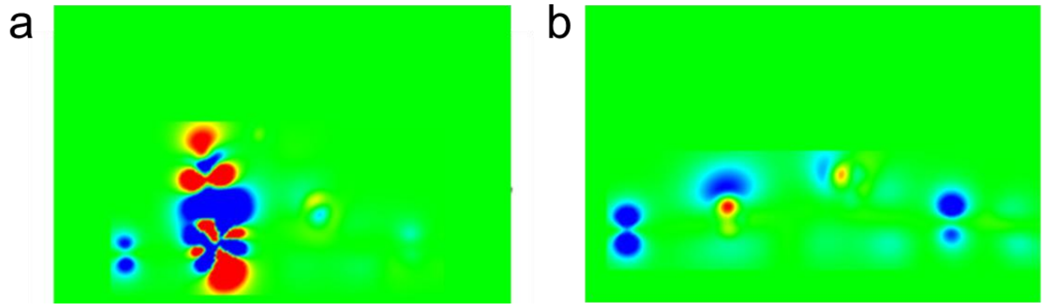

Figure S25. 2D projection of the charge density contour of (a) FeBi@CN-Li_2S_4 and (b) ZnBi@CN-Li_2S_4.

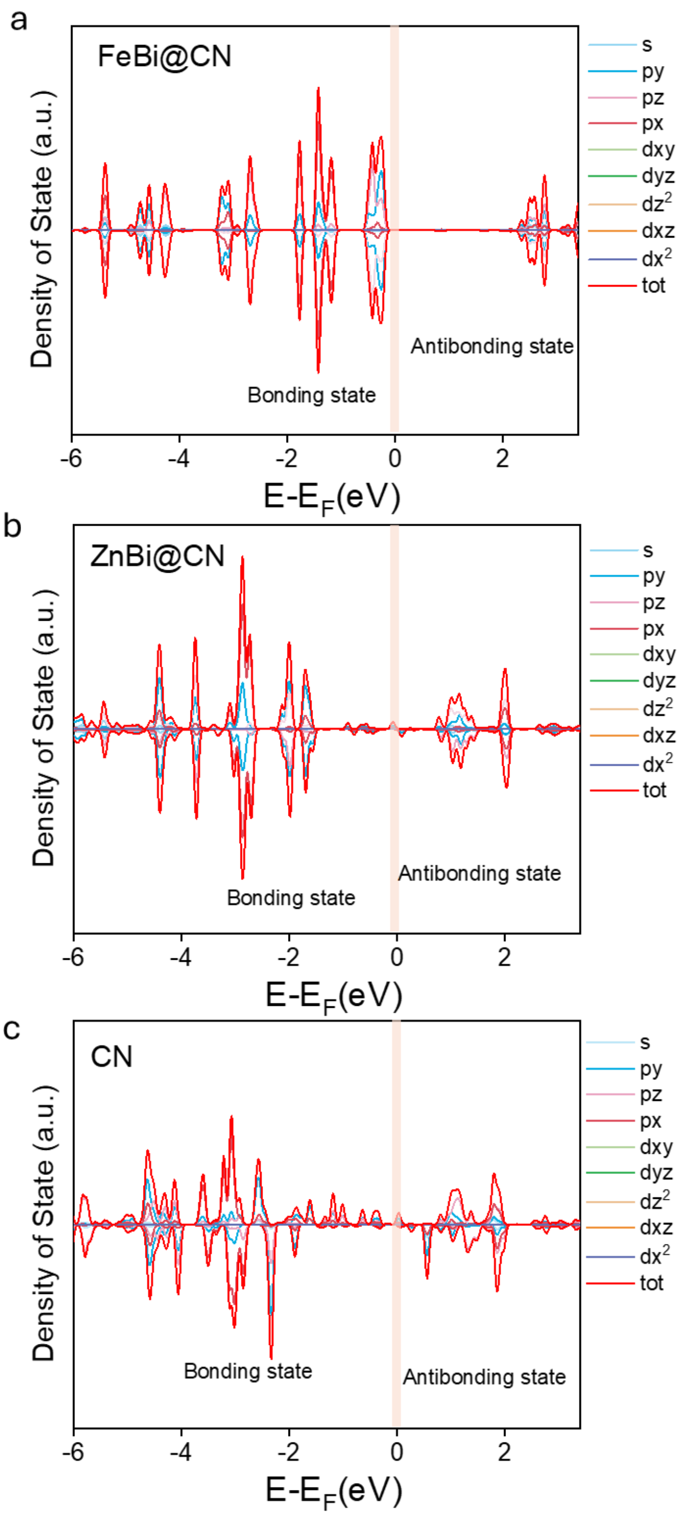

Figure S26. PDOS of bonding state orbital hybridization for Li_2S_4 on FeBi@CN, ZnBi@CN, and CN.

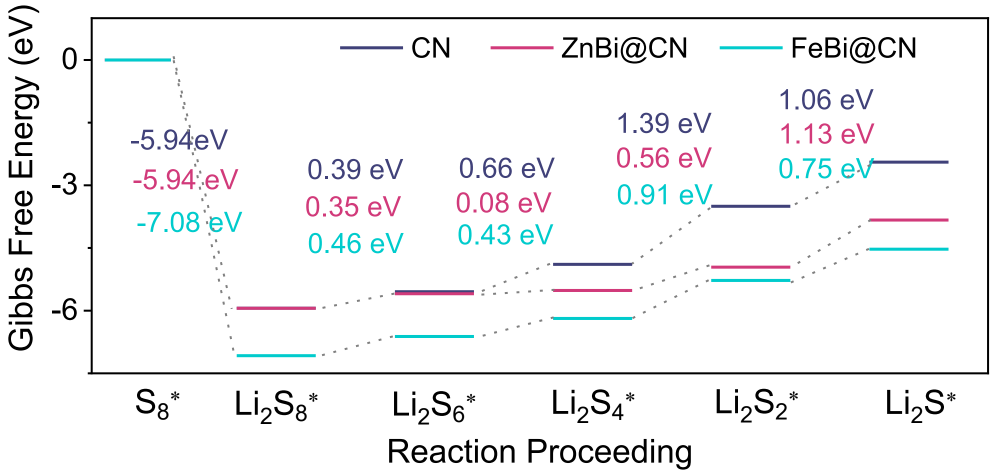

Figure S27. Gibbs free energy of S_8, Li_2S_8, Li_2S_6, Li_2S_4, Li_2S_2, and Li_2S on CN, ZnBi@CN, and FeBi@CN catalysts.

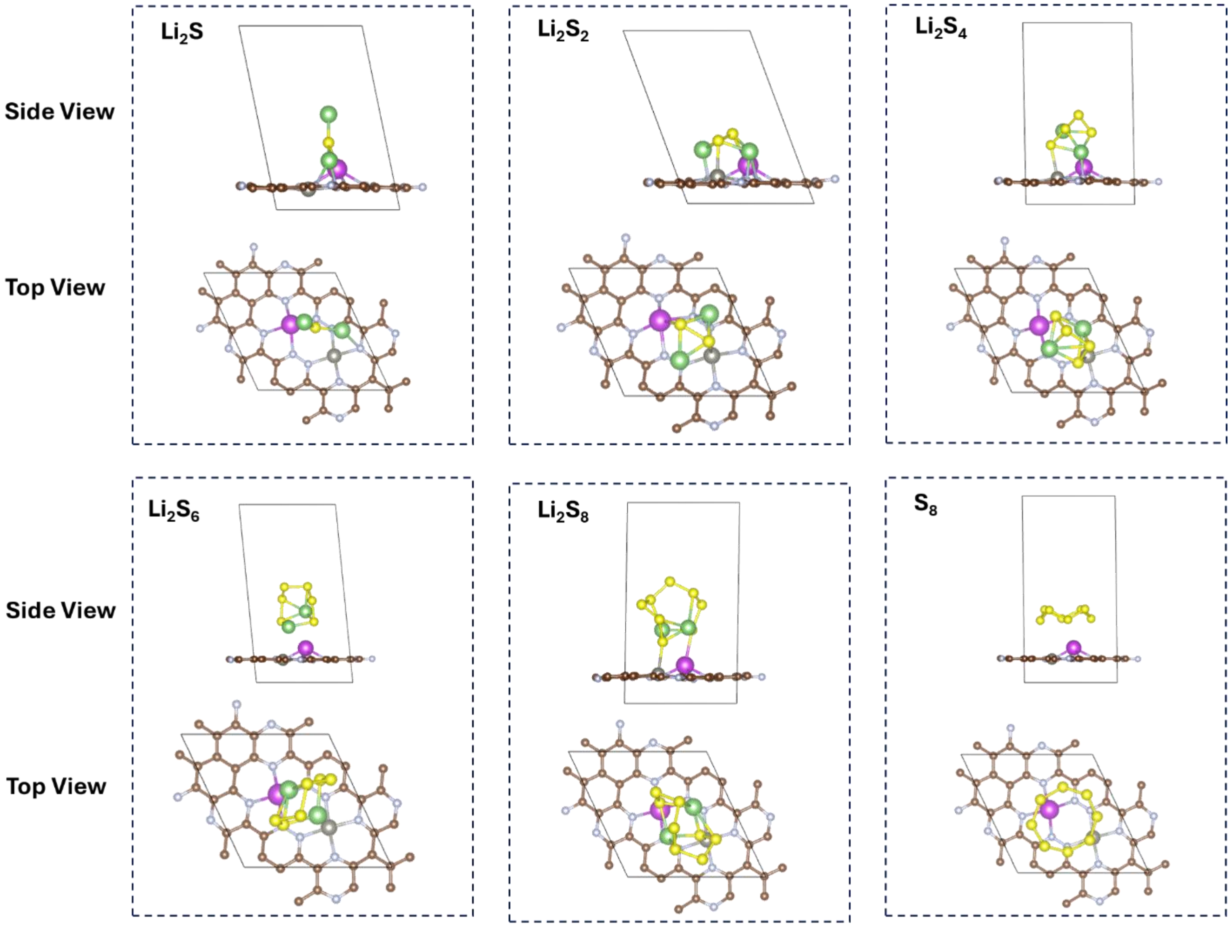

Figure S28. DFT optimized geometrical configuration of FeBi@CN with LiPS.

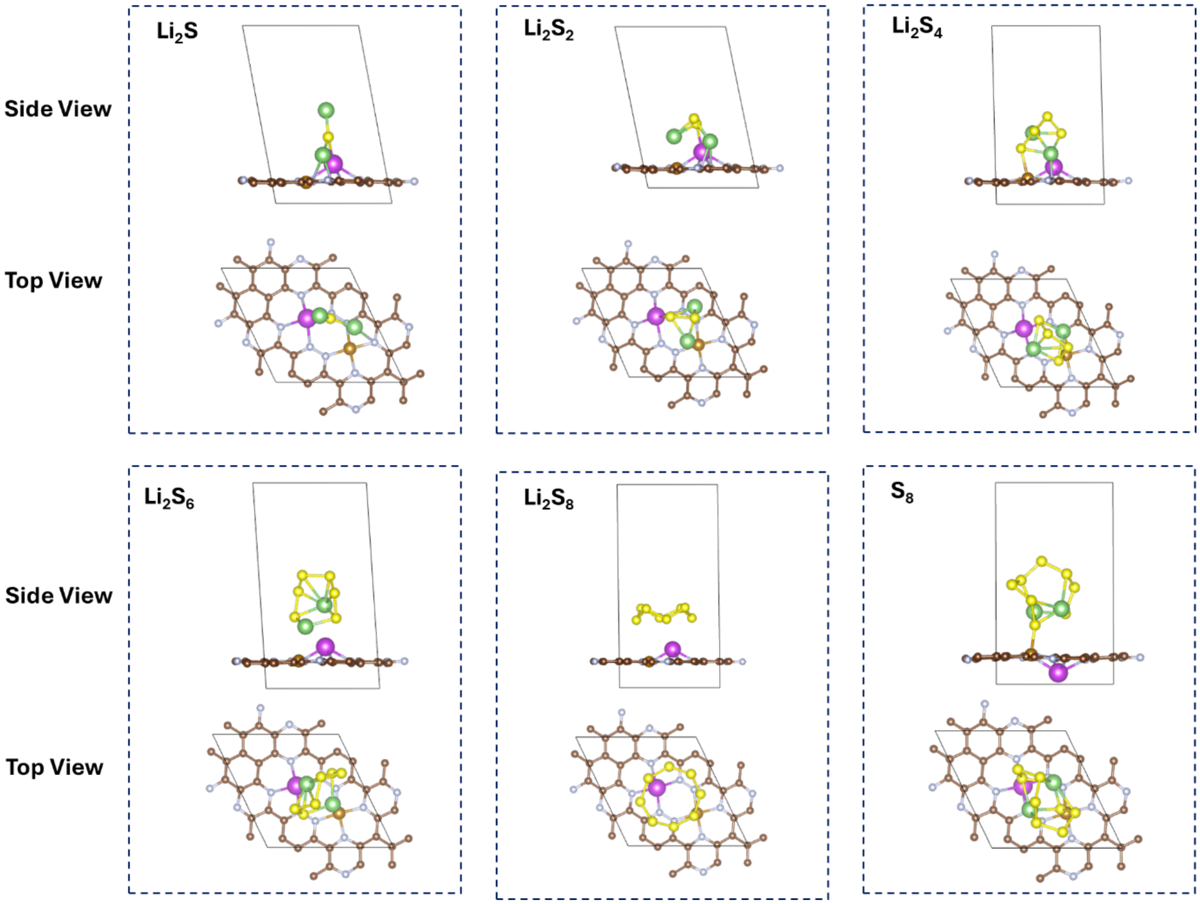

Figure S29. DFT optimized geometrical configuration of ZnBi@CN with LiPS.

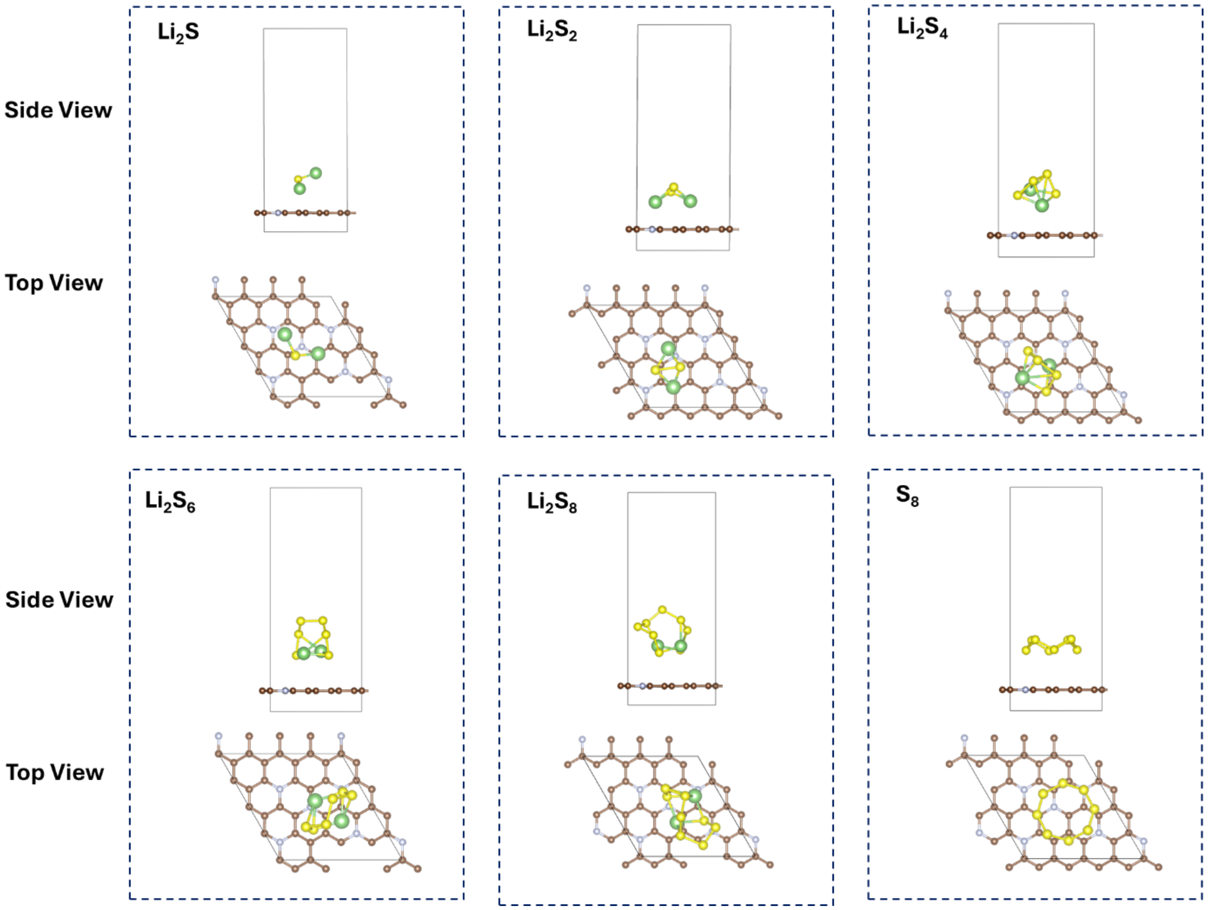

Figure S30. DFT optimized geometrical configuration of CN with LiPS.

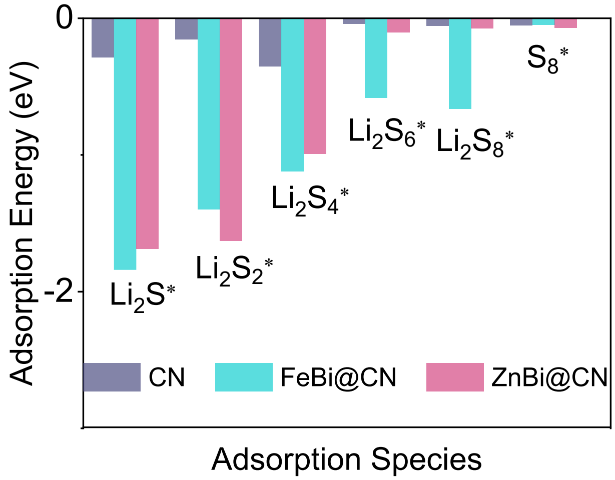

Figure S31. Adsorption energies of S_8, Li_2S_8, Li_2S_6, Li_2S_4, Li_2S_2, Li_2S on CN, ZnBi@CN, and FeBi@CN catalysts.

Table S1. Concentration of metal ions in various samples measured by ICP-OES.

Samples	TM Concentration (%)	RSD (%)	Bi Concentration (%)	RSD (%)
FeBi@CN	0.403	2.5	0.444	0.47
ZnBi@CN	0.442	1.5	0.492	0.87

RSD: Relative standard deviation

Table S2. Overpotential and square area of symmetrical cells CV curves of different catalysts.

Scan Speed	Overpotential peak II(V)		Charge (Coulomb)	
($mV\ s^{-1}$)	FeBi@CN	ZnBi@CN	FeBi@CN	ZnBi@CN
1	0.956	1.13	3.8383	2.9676
2	1.163	1.436	7.0759	5.7800
3	1.777	1.814	10.0846	8.0529
4	1.595	2.315	12.1123	8.6838
5	2.205	2.118	13.3560	7.6930
6	2.085	2.17	14.8977	7.2310
7	2.22	2.32	16.1908	7.8215
8	2.84	2.4	16.8898	8.4726
9	2.4	2.58	17.6569	8.8321
10	2.49	2.59	18.4445	8.9342

Table S3. voltage gap (ΔE) at different rates of different catalysts.

**Current Density**	**ΔE (V)**	**ΔE (V)**	**ΔE (V)**	**ΔE (V)**
	**FeBi@CN**	**ZnBi@CN**	**Bi@CN/S**	**SP/S**
0.1 C	0.17	0.14	0.16	0.23
0.2 C	0.18	0.19	0.20	0.22
0.5 C	0.21	0.25	0.33	0.30

**Current Density**	**ΔE (V)**	**ΔE (V)**	**ΔE (V)**	**ΔE (V)**
	**FeBi@CN**	**ZnBi@CN**	**Bi@CN/S**	**SP/S**
1 C	0.24	0.34	0.41	0.33
2 C	0.28	0.47	0.46	0.39
3 C	0.31	0.59	0.69	0.84
